# Integration of bioinformatic tools for the detection of SARS-CoV-2 co-infection cases

**DOI:** 10.1099/mgen.0.001604

**Published:** 2026-01-29

**Authors:** Adeliza Mae L. Realingo, Francisco Gerardo M. Polotan, Miguel Francisco B. Abulencia, Roslind Anne R. Pantoni, Jessel Babe G. Capin, Gerald Ivan Sotelo, Maria Carmen A. Corpuz, Neil Tristan M. Yabut, Saul M. Rojas, Ma. Angelica Tujan, Karen Iana Tomas, Ardiane Ysabelle Dolor, Czarina Christelle Alyannah Celis, Stephen Paul Ortia, Ezekiel A. Melo, Chelsea Mae M. Reyes, Elijah Miguel P. Flores, Anne Pauline A. Alpino, Aldwin Kim A. Penales, Kathlene Mae C. Medina, Joanna Ina Manalo, Timothy John R. Dizon, Katie Hampson, Sandeep Kasaragod, Joseph Hughes, Kirstyn Brunker

**Affiliations:** 1 Advanced Molecular Technologies Laboratory, Research Institute for Tropical Medicine, Department of Health, Manila, Philippines; 2 School of Biodiversity, One Health & Veterinary Medicine, University of Glasgow, Glasgow, UK; 3 MRC-University of Glasgow Centre for Virus Research, Glasgow, UK

**Keywords:** bioinformatics pipeline, co-infection, genomic surveillance, nucleotide mixtures, recombination, SARS-CoV-2

## Abstract

Co-infection with multiple severe acute respiratory syndrome coronavirus 2 (SARS-CoV-2) variants, though rare, may have clinical and public health implications, including facilitating variant recombination. Early detection of co-infections is, therefore, crucial. In this study, we report two probable cases of co-infection identified during routine genomic surveillance. Initially suspected as cross-contamination due to the presence of private mutations and nucleotide mixtures flagged by Nextclade and bammix, the samples were re-extracted and re-sequenced after workspace decontamination, yet the anomalies persisted. To investigate further, we developed a bioinformatics pipeline (Katmon) incorporating various tools such as Freyja, with lineage abundance results that illustrated the presence of multiple variants, and VirStrain, which confirmed inconsistent lineage assignments. We also visualized the alternative allele fractions for each lineage-defining mutation and amplicon, showing evidence of two variants, Delta and Omicron, co-existing within a single amplicon. Amplicon sorting effectively separated reads corresponding to the two variants, and the resulting consensus sequences aligned with their respective lineage assignments. These findings suggest that the first sample, PH-RITM-1395, involved a Delta–Omicron co-infection, while the second sample, PH-RITM-4146, probably contains both a co-infection and a recombinant variant. To further support the second sample’s recombinant nature, we employed sc2rf, which identified Delta–Omicron breakpoints. Retrospective analysis of 1,078 samples from July 2021 to July 2022, encompassing the period of co-circulation of different variants in the Philippines, flagged four additional co-infection cases, including Delta–Omicron and Beta–Omicron, suggesting a lower bound co-infection prevalence of 0.27% and 0.19%, respectively. Furthermore, the pipeline was used to test previously identified co-infections of different variants from different countries. Our findings underscore the critical importance of real-time genomic surveillance and advanced bioinformatics pipelines in detecting SARS-CoV-2 co-infections and variant recombination.

**Impact Statement**

This is the first report of severe acute respiratory syndrome coronavirus 2 (SARS-CoV-2) variants co-infection in the Philippines, which led to the development of a bioinformatics pipeline and enabled retrospective analysis of genomic surveillance sequences, uncovering additional co-infection cases. The pipeline was validated on simulated co-infections and publicly available sequences of confirmed co-infection cases from other countries.

## Data Summary

The raw FASTQ files of the co-infection samples are associated with the following accession in the Sequence Read Archive: SRR29688772, SRR29462594, SRR24282421, SRR24282418, SRR35941993 and SRR35941994. Their corresponding consensus FASTA sequences are deposited in Global Initiative on Sharing All Influenza Data (GISAID) with the following codes: EPI_ISL_12911897, EPI_ISL_19227219, EPI_ISL_9177619, EPI_ISL_12911895, EPI_ISL_12911898 and EPI_ISL_12703512. The simulation scripts, generated reads and the corresponding Katmon pipeline results are available in a repository, https://github.com/lanadelrea/simKatmon. The pipeline scripts are publicly available in the Katmon repository, https://github.com/lanadelrea/Katmon. All sequences processed for Philippine SARS-CoV-2 genomic surveillance, including those used in this study, are routinely uploaded to and are publicly available on the EpiCoV database of GISAID. The EPI Set of the retrospective samples analysed with the pipeline is found in Table S2. All supplementary material is uploaded on figshare DOI: https://doi.org/10.6084/m9.figshare.30608807 [1].

## Introduction

Since the detection of the first case of coronavirus disease 2019 (COVID-19) in March 2020, the Philippines has experienced distinct epidemic waves marked by surges in COVID-19-related hospitalizations and deaths. The emergence of new variants has underpinned such epidemiological patterns globally, emphasizing the critical importance of genomic surveillance. The Philippines has emerged as a leading contributor to severe acute respiratory syndrome coronavirus 2 (SARS-CoV-2) sequencing in Southeast Asia, demonstrating the country’s commitment to strengthening its capacity for genomic surveillance of infectious diseases. This effort has contributed to an enhanced understanding of transmission patterns and the association of distinct SARS-CoV-2 lineages with different phases of the epidemic in the Philippines [2,4]. Several waves of SARS-CoV-2 variants of concern (VOC), associated with increased transmissibility and immune escape, have been identified during the epidemic. The Delta variant was first detected in May 2021, causing a surge in cases and prompting lockdowns and community quarantine throughout the country. Then, in November 2021, the Omicron variant was initially detected and soon dominated, displacing Delta. Co-circulation of lineages has allowed recombination events, producing the recombinant lineages, such as the Omicron XBB and sub-variants, FLiRT variants and NB.1.8.1, which have been circulating more recently[5,8].

Co-infection occurs when an individual is simultaneously infected with two distinct variants of the virus, typically during periods when the two variants of concern are co-circulating. In co-infection cases, the presence of multiple variants in a subset of host cells is a potential environment for recombination to occur [9,11]. Epidemiological evidence from Andalusian genomic surveillance (January to May 2022) indicates a correlation between an increase in co-infection cases and a rise in circulating recombinant variants, with the unexpectedly high number of co-infections contributing to the emergence of new Delta–Omicron and Omicron–Omicron recombinants [12]. A study by Jackson *et al.* [13] described multiple independent recombination events involving UK lineages, emphasizing the importance of co-infections during periods of high viral prevalence, which can drive the emergence of new recombinant lineages. Recombination can lead to the emergence of a virus with a novel phenotype by combining genetic material from two distinct variants. This process can result in changes to key viral traits, such as increased transmissibility, immune evasion or altered disease severity [10,14]. If the newly formed recombinant virus gains an advantage, it may spread more efficiently in the population, potentially leading to further outbreaks [15,16].

A recent study in the Philippines investigating the emergence of the recombinant lineage XBC identified one sample, collected in March 2022, with an allele fraction pattern and within-host mutations indicative of an active co-infection [17]. Since co-infection is a prerequisite for recombination, this suggests that co-infection cases were already present in the Philippines by early 2022. Genomic surveillance data from the country have also identified XBC sequences from as early as January 2022, a period when Delta was being replaced by Omicron. Phylogenomic methods by Turakhia *et al.* [16] identified 589 recombination events in 1.6 million samples publicly available in May 2021. While this represents a relatively low rate on a per-sample basis, it is significant in the context of viral evolution, particularly during periods of high viral prevalence. This emphasizes the importance of timely detection and analysis of recombinant lineages to pinpoint their emergence and understand their potential for enhanced epidemiological or phenotypic properties [10,16].

Co-infections are rarely reported, but such cases can have important clinical and epidemiological significance, potentially contributing to increased disease severity and viral immune escape[9,18]. A study by Rockett *et al.*[11] investigated two immunocompromised individuals who experienced co-infection of the Delta and Omicron variants. In one case, the patient was initially infected with Omicron and later superinfected with Delta before being admitted to the hospital. Another case study by Samoilov *et al.* [19] reported an elderly patient who was hospitalized with worsening symptoms that ultimately led to death. Genomic analysis revealed that the patient was infected with two distinct SARS-CoV-2 strains from different phylogenetic clades, GH and GR. Notably, the relative abundances of these lineages differed between the first and last swabs, taken just eight days apart. The detection of co-infection cases is likely underestimated [20,21], and it is, therefore, crucial to detect these cases during genomic surveillance as early as possible for clinical management, treatment strategies and implementing appropriate public health measures.

To accurately detect co-infection cases, it is essential to consider several factors during the sequencing process. Primer amplification bias of some genomic regions of specific variants, contamination during the sequencing process (which might be the actual cause of nucleotide mixtures in the sample), and insufficient bioinformatics methods to systematically detect co-infections can all influence the ability to distinguish true co-infection cases from within-sample noise [22]. The sample needs to be sequenced at sufficiently high depth to identify and characterise co-infection of different VOCs [9]. As variants evolve, both sequencing procedures and bioinformatics tools need critical quality control measures to determine the presence of any co-infection, recombination and contamination in the sequence data. Currently, there are limited tools for identifying and describing SARS-CoV-2 co-infections of known variants. Rockett *et al.* [11] were able to flag heterozygous calls in sequences from epidemiologically unrelated patients using a bioinformatic quality control system, similar to the observations that prompted further investigation in our study. Molina-Mora *et al.*[23], developed a metagenomic pipeline to identify co-infections of distinct SARS-CoV-2 variants as part of their genomic surveillance, while Bolze *et al.*[9] examined allele fraction patterns to distinguish co-infection and recombinant samples. Taken together, these studies highlight that detecting SARS-CoV-2 co-infections often requires a combination of complementary approaches, which may vary on a case-by-case basis.

Co-infections may have been occurring in the Philippines as early as March 2022 [17]. Here, we investigate two samples, collected in May 2022 and April 2023, flagged as possible Delta and Omicron co-infections during routine genomic surveillance efforts at the Research Institute for Tropical Medicine (RITM). The samples prompted the development of a robust automated pipeline, integrating various independent tools, to diagnose co-infection cases, and we retrospectively quantified their prevalence in a large cohort of samples sequenced during the period where Delta, Omicron and other variants co-circulated in the Philippines. Additionally, we validated the pipeline against a range of samples from previous studies from different countries and test its limits of detection using simulated sequences and semi-synthetic mixtures.

## Methods

### Sample collection, RNA inactivation and extraction

Samples obtained from naso- and oro-pharyngeal swabs of COVID-19-positive patients were endorsed by the Epidemiology Bureau (EB) to the RITM for sequencing as part of the routine SARS-CoV-2 genomic biosurveillance in the Philippines. RNA was inactivated and extracted using MagMax™ Viral/Pathogen Nucleic Acid Isolation Kit (A42352, Thermo Fisher) in the Kingfisher flex purification system. This protocol used 200 µl of clinical specimens and a final elution volume of 80 µl.

As part of the sequencing protocol, the identifiers used for each sequence were assigned in the laboratory during processing and cannot be directly traced to patients from whom the samples were collected. Among all the samples, we further investigated two sequences, PH-RITM-1395 and PH-RITM-4146, as possible co-infection cases because of their inconsistent lineage assignment and unusual number of private mutations flagged by Nextclade (v2.14.1). These two samples were collected at different time points and regions of the Philippines. Since the specimens were endorsed by EB, no serology or reverse transcription quantitative real-time PCR reverse transcription quantitative real-time PCR (RT-qPCR) testing for other respiratory pathogens was performed.

### Whole-genome sequencing using Oxford Nanopore Technology

** **Sequencing libraries were prepared using the ARTIC LoCost sequencing protocol [24]. The cDNA was prepared by reverse transcription using LunaScript^Ⓡ^ RT SuperMix Kit (E3010, NEB) and ARTIC primers v4+4.1 were used with Q5^Ⓡ^ Hot Start High-Fidelity 2X Master Mix (M0494, NEB) to amplify the whole genome in a multiplex PCR reaction, followed by purification of the DNA using 0.9X AMPure XP beads (A63881, Beckman Coulter). Sample concentration was checked using a Qubit 4 fluorometer, with acceptable concentrations of >10 ng ul^−1^. To prepare for Oxford Nanopore Technology (ONT) whole-genome sequencing, the amplicons were library prepared and barcoded using Ultra II reagents (E7546 and E7595, NEB) and ONT Native Barcoding Kit (EXP-NBD196), cleaned up with 0.4X AMPure XP beads, and the adapters ligated using the NEBNext Quick Ligation Module (E6056, NEB). The final libraries were prepared using the ONT Ligation Sequencing Kit (SQK-LSK109) and loaded into an R9.4.1 flow cell (FLO-MIN106D, ONT). Sample PH-RITM-1395 was sequenced on a GridION with high-accuracy basecalling for 72 h, while sample PH-RITM-4146 was sequenced on a MinION Mk1B device with fast basecalling for 48 h. The two samples investigated were about a year apart, and the protocols were optimized during that period. Given the limited resources in our laboratory, we determined that 48 h of sequencing combined with fast basecalling was sufficient to achieve the required depth and quality of reads for downstream SARS-CoV-2 read analysis. In addition, due to the unavailability of the GridION platform at the time for sequencing the second sample, the MinION Mk1B was used instead.

### Detection of SARS-CoV-2 variant of concern and pango-lineage

Following the RITM routine process for SARS-CoV-2 genomic surveillance, we used the ARTIC bioinformatics standard protocol for SARS-CoV-2 implemented in the ncov2019-artic-nf nextflow workflow (Artic Network, 2020) to generate a consensus sequence and assign a pango-lineage to the samples. Basecalling of raw ONT FAST5 data was performed using guppy software (v5.1.13). Raw FAST5 and FASTQ files were transferred to the RITM high-performance computing server. The workflow performs demultiplexing of samples by barcodes, read filtering by length of 400–700 bp and mapping of basecalled reads to raw FAST5 signal data. FASTQ reads are then aligned to the SARS-CoV-2 reference genome using minimap2. Primers are trimmed from aligned reads, and sequences of aligned reads falling outside primer boundaries are soft-clipped. Variants are called using Nanopolish for each primer pool separately. Variant calls are merged into a single VCF file and then filtered with the criteria of >20× depth and a minimum of 50% supporting reads from each strand. Regions of the genome covered with <20× depth are masked, and variant calls are introduced to the regions with >20× depth to produce the consensus sequence. Pangolin tool, including Scorpio (v0.3.17), with default UShER-1.2 as lineage assignment engine, was used to assign the consensus sequence to a SARS-CoV-2 lineage, and Nextclade (v2.14.1) was used for quality control. As part of genomic surveillance, all the sequences having greater than 70% genome coverage, including the samples investigated in this study, were shared publicly with the Global Initiative for Sharing All Influenza Data (GISAID).

### Detection of Omicron and Delta co-infection

A close examination of the sequence with anomalous quality control metrics was performed, including inspection of the quality of the raw reads, the consensus sequence, variant calls and read-level alignment. FastQC (v0.11.9) was used to evaluate the quality of basecalled FASTQ reads. The Nextclade (v2.14.1) web server was used to evaluate the quality of the consensus sequence, including metrics such as percent coverage, depth, unexpected private mutations, frameshifts and stop codons. The bammix (v1.0.0) tool was used to visualize and identify all the genome positions with heterogeneous nucleotide bases from aligned reads, i.e. not a single dominant nucleotide base but a mixture. The covSPECTRUM and CoVariants web resources were used to identify the list of single-nucleotide variants (SNVs) that were distinct between the mixed lineages suspected to be present in the same sample. Multiple SNVs that were located near enough each other and could be spanned by reads that are 400–700 bp long were inspected in the alignment BAM file using Tablet software (v1.21.02.08) [25]. Individual reads in the Tablet containing the expected SNVs of one of the two lineages but not the other were identified as supporting evidence that multiple lineages were present in the sample. We used VirStrain (v1.17) to confirm lineage inconsistencies from the FASTQ files and Freyja (v1.5.1) to estimate lineage abundance from the BAM files.

We then looked at the alternative allele fraction (AAF), using custom Python scripts adapted from Bolze *et al.* [9], to determine the number of reads supporting each VOC. AAF is the ratio between the number of reads supporting an alternative allele or mutation and the total number of reads covering the corresponding position. Then, we illustrated the average AAF of Delta and Omicron per amplicon by first filtering high-quality reads from the BAM files and determining the proportion of lineage-defining mutations. The range of each amplicon was defined by a BED file from the ARTIC Network’s version 4.1 primer scheme, available at https://github.com/artic-network/artic-ncov2019. Lastly, using the tool samjdk from jvarkit [26], we separated the Omicron and Delta reads, created independent FASTA sequences from the sorted reads and determined their lineage assignment.

### Pipeline development

**Fig. 1.**
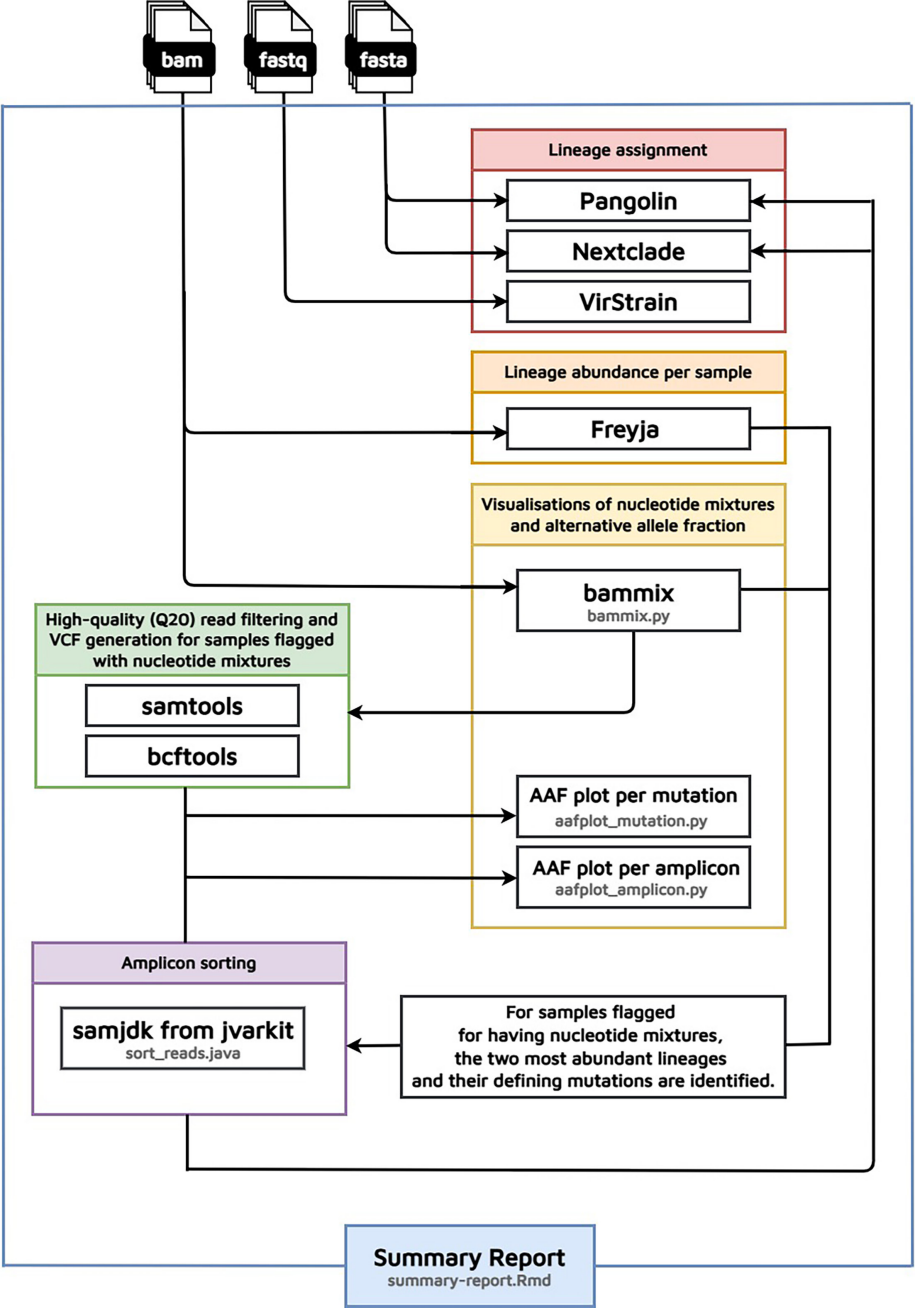
Flow of the Katmon pipeline. The pipeline developed to analyse samples that were flagged as probable co-infection used various tools for lineage assignment, lineage abundance, nucleotide mixtures visualizations and amplicon sorting. Some of the steps used custom Python, Java and R scripts. The pipeline outputs all the results from the tools in sorted folders and creates a summary report.

Following the steps of the manual investigation of the two samples, we developed the Katmon pipeline (Fig. 1). The pipeline is written using Nextflow, allowing multiple processes to run in parallel for efficient analysis [27]. It is publicly accessible at https://github.com/lanadelrea/Katmon. As input, the pipeline takes BAM, BAM index, FASTQ and FASTA files produced during genomic surveillance. The use of the different file types is primarily driven by the varying input requirements of different tools and enables us to examine the sample from multiple perspectives. The pipeline consists of six key steps: (1) lineage assignment using the tools Pangolin, Nextclade, and VirStrain; (2) lineage abundance estimation using Freyja; (3) visualization of nucleotide mixtures with bammix; (4) plotting of alternative allele fraction of filtered high quality reads (Q20); and (5) amplicon sorting of reads using the tool samjdk from jvarkit. The input for the amplicon sorting step is the tabulated lineage-defining mutations of the two most abundant lineages detected from Freyja. Finally, the pipeline creates a comprehensive summary report that integrates all tables and plots from these processes.

Having a method that independently utilizes different tools and then assesses their agreement provides a significant advantage because each tool possesses its own limitations. Since Pangolin [28] and Nextclade [29] assign lineages by only using the consensus FASTA, we cannot directly confirm the presence of co-infection in the sample. We then use bammix [30] to determine whether these flagged positions contain nucleotide mixtures; however, it cannot determine the specific variants to which the mutations belong to. Then, to compare lineage assignments based on raw FASTQ file reads, we use VirStrain, a strain-level analysis and haplotype reconstruction tool. Its limitations are that it cannot accurately detect low-abundance viral strains from very short reads (depth <10×, read length <100 bp) and relies on a custom database that must be updated whenever new variants emerge [31]. In contrast, Freyja, using the BAM files as input, can resolve lineages even at 5% abundance but requires greater sequencing depth to obtain a more accurate estimate of the true mutation frequency in the samples [32]. Freyja is reported to show relative abundance estimates with high accuracy compared to other tools [33]. The Katmon pipeline integrates these multiple analytical tools, examining data from different perspectives to allow the identification of congruent signals, thereby providing a reliable and conservative estimate of co-infection.

The pipeline was initially designed to report possible Delta and Omicron co-infections. We subsequently expanded its capability to detect potential co-infections for any SARS-CoV-2 variants, including co-infections of newer variants and cases involving two lineages of the same clade. To achieve this, we leveraged the ability of Freyja to identify the two most abundant lineages in a sample and retrieve their lineage-defining mutations. These mutations are then used to plot alternative allele fractions and perform amplicon sorting, which improves on the previous method of only sorting reads specific to Delta and Omicron. We set the default threshold of bammix for detecting minor alleles to 20%. This threshold can be adjusted by the user as needed.

### Validation using *in silico*-generated reads, semi-synthetic mixed reads, and publicly available co-infection sequences

To evaluate the pipeline’s ability to detect low-abundance mixtures and co-infections involving lineages from the same or different clades, we performed *in silico* simulations using ww_simulations (https://github.com/CFSAN-Biostatistics/ww_simulations)[33] and generated Illumina reads mimicking co-infections. Simulated datasets included randomized lineage combinations at varying proportions (2: 98, 5 : 95, 10 : 90, 20 : 80 and 50 : 50). Three mixture types were tested: Delta–Omicron (AY.107 and BA.2.3), Delta–Delta (AY.132 and AY.63) and Omicron–Omicron (BA.1.10 and BA.2.5), allowing us to assess the pipeline’s performance in detecting not only distinct variant co-infections but also those involving a variant and its subvariant. The simulation scripts, generated reads and the corresponding Katmon pipeline results are available in a public repository, https://github.com/lanadelrea/simKatmon.

To further evaluate performance with long-read data, we simulated ONT reads using Badread (https://github.com/rrwick/Badread)[34] under the nanopore2023 and nanopore2020 error models, generating Delta–Omicron mixtures across the same proportions (2 : 98 to 50 : 50) and varying error rates (2%, 5%, 10% and 20%). In addition, we generated mixed reads from pure Delta (SRX26994353) and Omicron BA.2 (SRX27000564) ONT reads to create semi-synthetic co-infections of Delta–Omicron at varying proportions using the tool rasusa (https://github.com/mbhall88/rasusa)[35].

Finally, the pipeline was tested on publicly available SARS-CoV-2 co-infection datasets from confirmed cases [9,12,36], representing sequences from France, Brazil, Australia, Spain and Norway, respectively (Table S1, available in the online Supplementary Material).

## Results

### Initial discrepancies in lineage assignment

Two samples processed for routine SARS-CoV-2 genomic surveillance were flagged for additional quality control checks due to inconsistencies in their lineage assignments and flagged by Nextclade for having multiple private mutations (Table 1, Fig. S1).

**Table 1.** Lineage assignment using Nextclade, Pangolin and Scorpio Notably, Nextclade documentation states that sequences with an unusually high number of private mutations are often flagged as potentially erroneous, possibly due to contamination, co-infection or recombination events [29].

Sample		Coverage	Nextclade	Pangolin	Scorpio	Note
**PH-RITM-1395**	First sequencing	99.6%	B.1.617.2 (Delta)	B.1.1.529 (Omicron)	Probable Omicron (unassigned)	UShER placements: B.1.1.529 (1/1); Scorpio found insufficient support to assign a specific lineage
	Replicate	91.9%	B.1.617.2 (Delta)	B.1.1.529 (Omicron)	Delta (B.1.617.2-like)	UShER placements: B.1.1.529 (1/1); scorpio lineage B.1.617.2 conflicts with inference lineage B.1.1.529
**PH-RITM-4146**	First sequencing	94.3%	B.1.617.2 (Delta)	AY.1 (Delta)	Delta (B.1.617.2-like) +K417 N	UShER placements: AY.1 (2/4), AY.122 (1/4), B.1.617.2 (1/4)
	Replicate	99.5%	B.1.617.2 (Delta)	AY.1 (Delta)	Delta (B.1.617.2-like) +K417 N	UShER placements: AY.1 (2/4), AY.122 (1/4), B.1.617.2 (1/4)

For sample PH-RITM-1395, the consensus sequence had a coverage of 99.57% and was initially identified as B.1.1.529 (Omicron) by the Pangolin tool, while Scorpio determined the sample to be a probable Omicron. This was discordant with Nextclade’s lineage assignment of B.1.617.2 (Delta). UShER (Ultrafast Sample placement on Existing tRees) placement noted that this sequence had insufficient support to be assigned to a specific lineage. Meanwhile, the sample PH-RITM-4146 had an initial coverage of 94.3% and was designated by Pangolin as AY.1 (Delta) and by Nextclade as B.1.617.2 (Delta). The Scorpio call also determined the sample as Delta B.1.617.2-like with an additional K417N mutation, characteristic of a Delta plus variant (Table 1).

### Contamination ruling and re-sequencing confirmation

Although collecting multiple samples from the same patient would have been ideal to rule out contamination, this was not possible in our case. Instead, we re-extracted and re-sequenced the samples to confirm the results and eliminate contamination as a factor. Upon doing so, we confirmed that the flagged private mutations persisted, thus ruling out the possibility of cross-contamination.

Further checks for cross-contamination in the wet lab protocol include a thorough investigation of the negative extraction control (NEC). The NEC is checked to ensure that its Qubit concentration is below 1 ng µl^−1^. When the Qubit concentration of the NEC exceeds the acceptable value, it is subjected to qPCR to check for amplification of the *E* and *N* genes. If the threshold cycle (Ct) values of the NEC are <40, the entire batch of samples is re-extracted. In the case of PH-RITM-1395, the NEC had a Qubit concentration of 0.4 ng µl^−1^, while the NEC of PH-RITM-4146 had a Qubit concentration of 0.120 ng µl^−1^. The samples had NECs passing the quality control, and, therefore, cross-contamination was ruled out.

Moreover, as part of the acceptance criteria for sequencing, the Ct value of the specimens was recorded. For the first sample, the Ct values were 19.4 for the *N* gene and 17.2 for the *E* gene. For the second sample, the Ct values were 30.82 for the *N* gene and 19.58 for ORF. These Ct values were low (<40), implying high source viral load that would be less susceptible to contamination, indicating suitability for whole-genome sequencing (WGS).

**Fig. 2.**
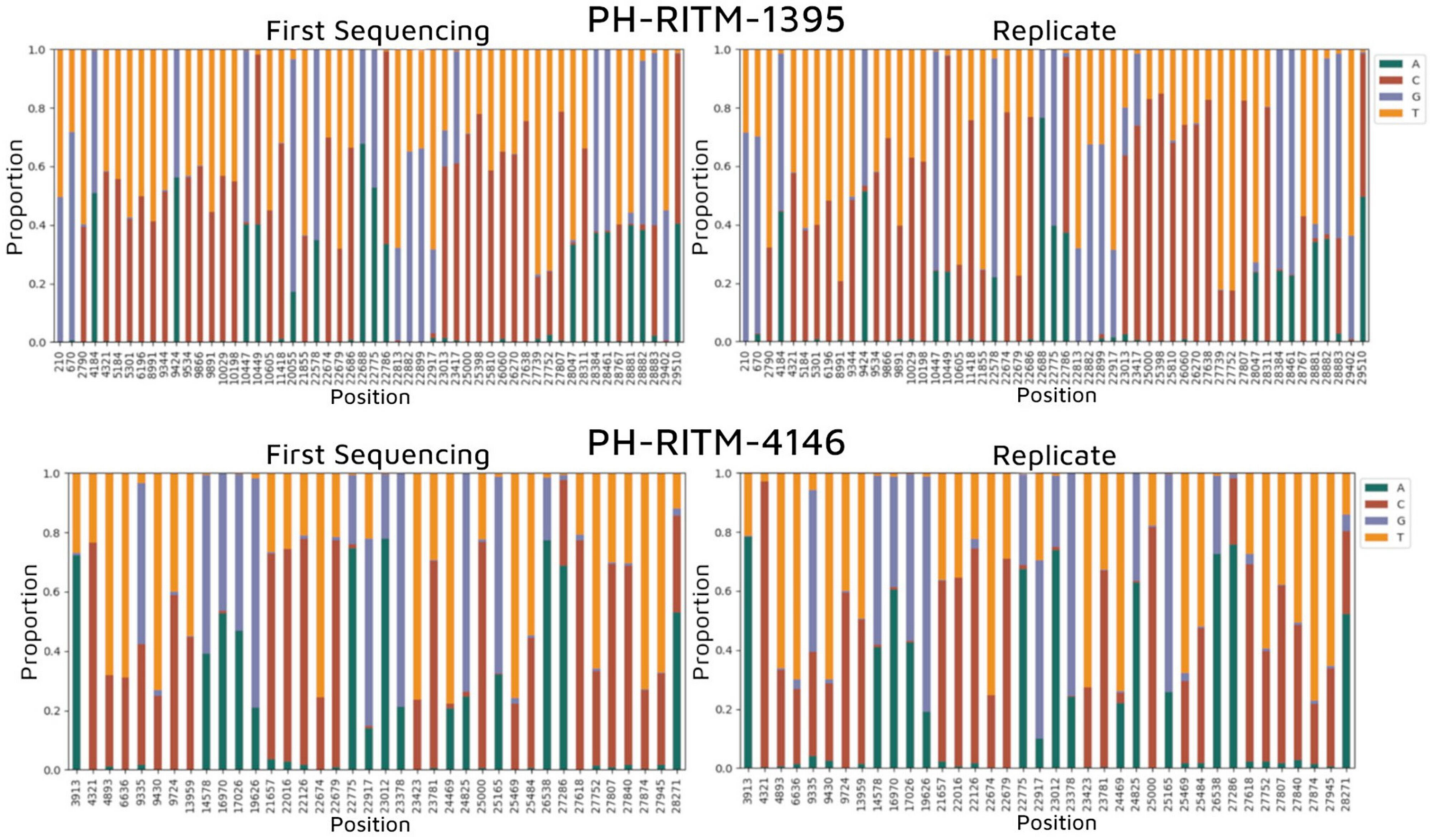
Genomic positions flagged by bammix having nucleotide mixtures for each sample. Shown are all sites with single-nucleotide variants (SNVs) or ambiguous base calls in the consensus assemblies of PH-RITM-1395 and PH-RITM-4146.

We then used bammix [30], which flagged multiple sites with nucleotide base mixtures exceeding our default threshold of 20% minor allele in both samples, as shown in Fig. 2. Multiple sites containing heterogeneous bases crossing the threshold in both replicates of each sample are also apparent. To validate these findings, we manually inspected the aligned sequence reads at each position using Tablet software [25] and observed the presence of a mixture of nucleotides on the flagged positions. It was apparent that the nucleotide mixtures persisted in the sequencing replicates for both samples. Since we ruled out cross-contamination as the source of lineage assignment discrepancies, we conducted a more thorough investigation for possible co-infection of the two VOCs Delta and Omicron using additional bioinformatics tools.

### Confirmation of co-infection

Observation of the nucleotide positions flagged by bammix and examination of overlapping SNVs and mutations support our hypothesis that the samples were from individuals co-infected with Delta and Omicron. To confirm co-infection, we further analysed the sample using VirStrain [31] and Freyja [32] and investigated the allele fraction of sequencing reads that mapped to mutations unique to each variant of concern.

#### VirStrain

Both samples were run against a custom VirStrain database created from the downloadable multi-fasta file of variants of interest, representative sequences from GISAID, and the European Nucleotide Archive COVID-19 Data Portal. VirStrain identifies the closest relative lineage in the database and can detect two strains with the same clades while providing accurate abundance predictions [31]. For PH-RITM-1395, results from VirStrain were consistent with the lineage assignment from both Pangolin and Nextclade: B.1.617.2 (Delta) as the most probable strain, with BA.2 (Omicron) as the other possible strain. Meanwhile, for PH-RITM-4146, the most probable strains were AY.4 (Delta) and BA.3 (Omicron), discordant with the results from the two lineage assignment tools.

#### Freyja

We then used Freyja, a tool designed to estimate the relative abundances of SARS-CoV-2 lineages in mixed samples from BAM files aligned to the Hu-1 reference. This tool uses lineage-specific mutational barcodes derived from the UShER global phylogenetic tree to ensure non-negative values and that the lineage proportions sum to one [32].

**Fig. 3.**
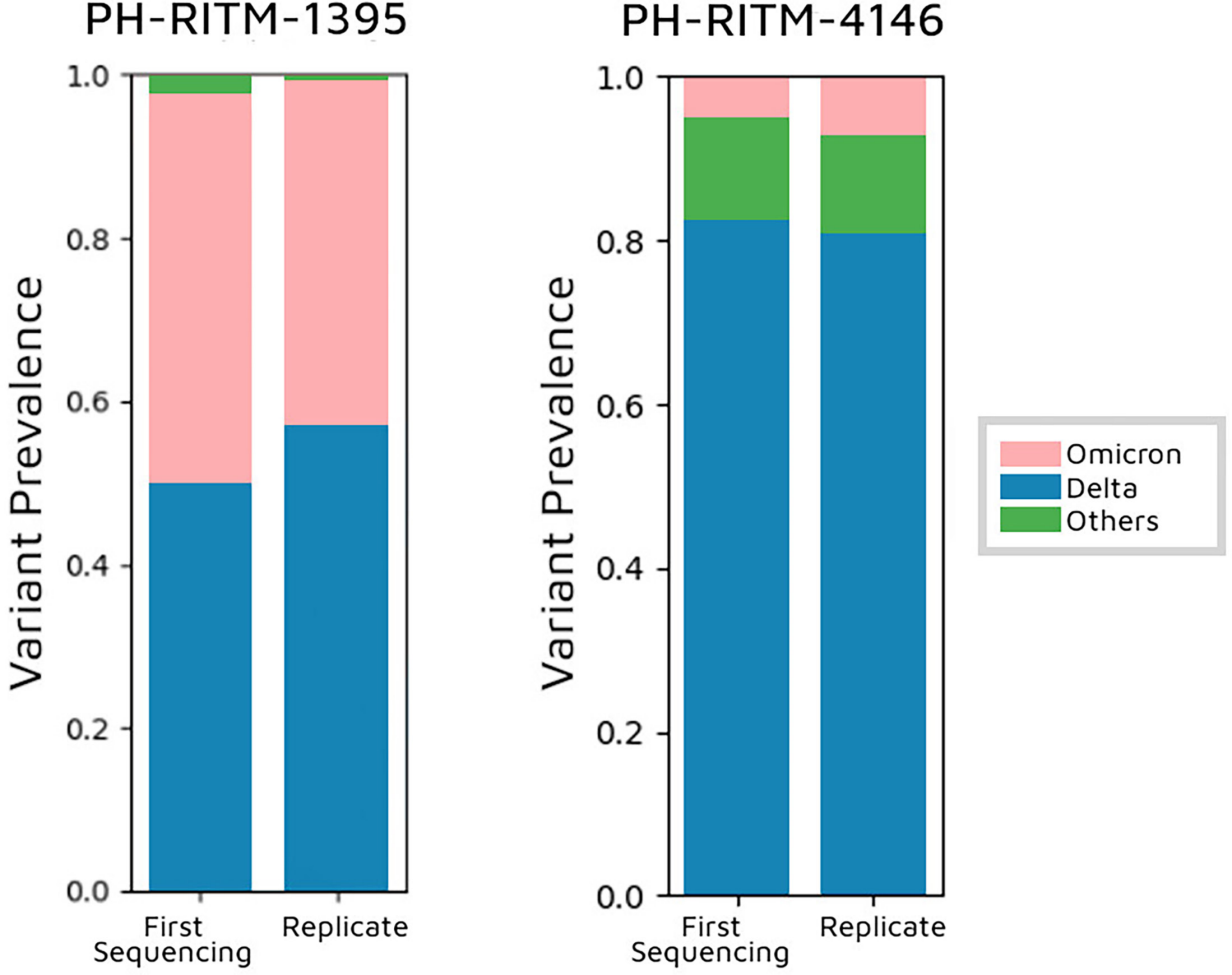
Freyja variant prevalence results. Variant frequencies for samples PH-RITM-1395 and PH-RITM-4146 are shown for the initial and replicated sequencing runs, as estimated by the Freyja tool. The category ‘Others’ (shown in green) represents lineages with less than 1% relative abundance that were grouped.

For the initial sequence of PH-RITM-1395, the abundances of Delta and Omicron are almost the same for the first and replicate sequences, and other variants were reported, but at low levels. The estimated lineage abundance of Delta and Omicron was 49.81% and 48.09%, respectively. The replicate sequence had a slight increase in Delta abundance, 56.83% as compared to Omicron with 42.55%. (Fig. 3). Fischer’s exact test was used to compare the proportions of Delta and Omicron reads between the first and replicate sequencing runs, and no significant difference was observed between the two runs (*P*-value=1.0). This indicates that the relative proportions of Delta and Omicron remained consistent, supporting that the observed mixed variant pattern reflects a co-infection rather than contamination.

For sample PH-RITM-4146, most reads were classified as Delta, with a smaller proportion identified as Omicron. Notably, Freyja detected a higher prevalence of ‘Others’ compared to Omicron. The ‘Other’ category was primarily composed of recombinant lineages: XAC and XZ, both derived from Omicron sublineages BA.2 and BA.1; XD, a recombinant of B.1.617.2 (Delta) and BA.1 (Omicron); and XAY, a recombinant of AY.45 and BA.4/BA.5 (Fig. 3). These findings suggest that sample PH-RITM-4146 may contain both Delta and Omicron variants, as well as one or more possible recombinant variants.

#### Comparative allele fraction analysis

**Fig. 4.**
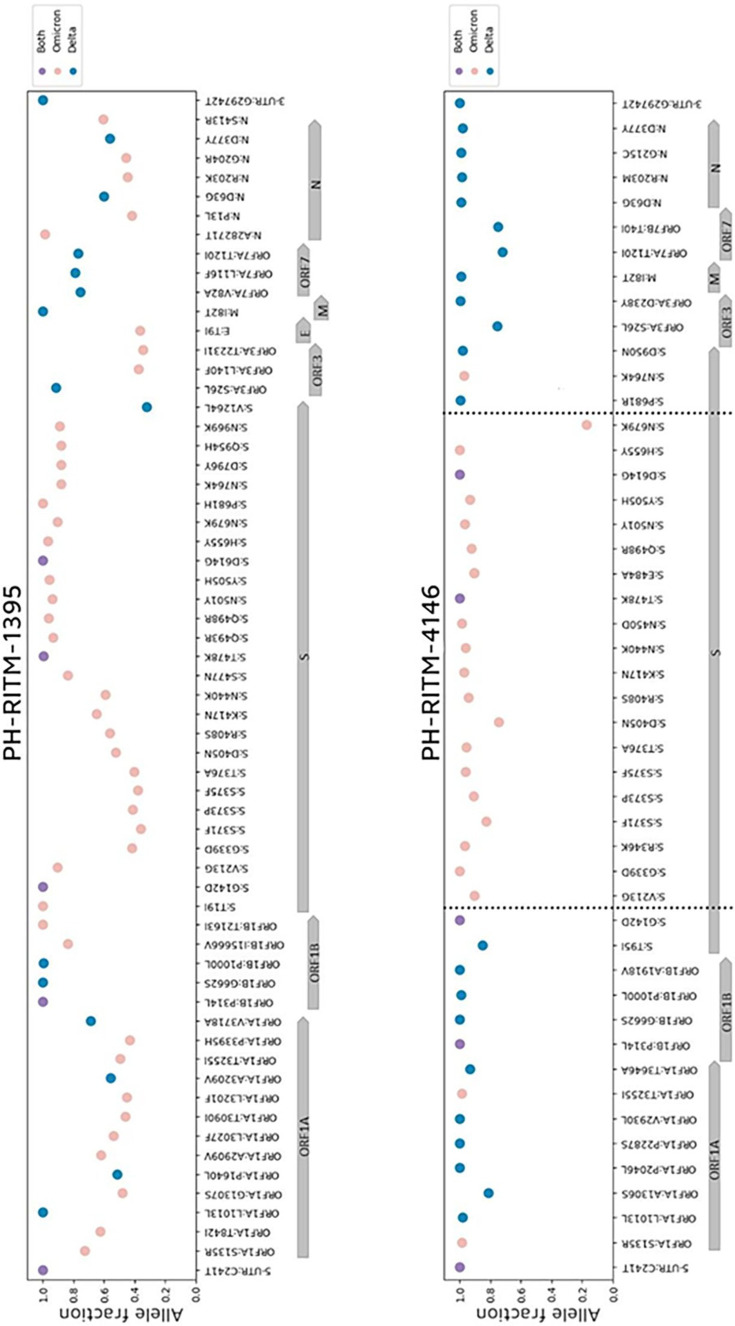
Alternative allele fraction of PH-RITM-1395 and PH-RITM-4146 for each lineage-defining mutation across the SARS-CoV-2 genome. Delta, Omicron and shared mutations are represented by blue, pink and purple, respectively. The vertical dotted lines in PH-RITM-4146 represent the inferred potential breakpoints in the recombinant lineage found in the sample.

From cov-spectrum.org, we listed the established mutations that are shared and distinct to B.1.617.2 (Delta) and B.1.1.529 (Omicron). Delta has 74 lineage-defining mutations, while Omicron has 51. Additionally, four amino acid changes are shared between the two, namely, ORF1b:P314L, S:S477N, S:T478K and S:D614G. These mutations were tabulated in a TSV file, which served as our lookup table for plotting the alternative allele fractions similar to the co-infection analysis described by Bolze *et al.* [9]. We utilized custom Python scripts illustrating the alternative allele fraction of the mutations across the SARS-CoV-2 genome of both samples (Fig. 4). Only high-quality reads (Q 20) were used to create the allele fraction plots. Mutations shared between the two variants have an allele fraction equal to one, as expected, since the allele is found in all of the reads in that position.

As shown in Fig. 4, the presence of both Delta and Omicron defining mutations in PH-RITM-1395 supports the hypothesis that this sample represents a co-infection case, as the mixed alleles exhibit fractions below one, which is a pattern consistent with co-infection [9]. In contrast, sample PH-RITM-4146 reveals Delta and Omicron lineage-defining mutations in distinct genomic regions, with allele fractions close to one and a sudden drop in allele fraction at the Spike mutation N679K, a pattern characteristic of recombinants also described by Bolze *et al.* [9].

**Fig. 5.**
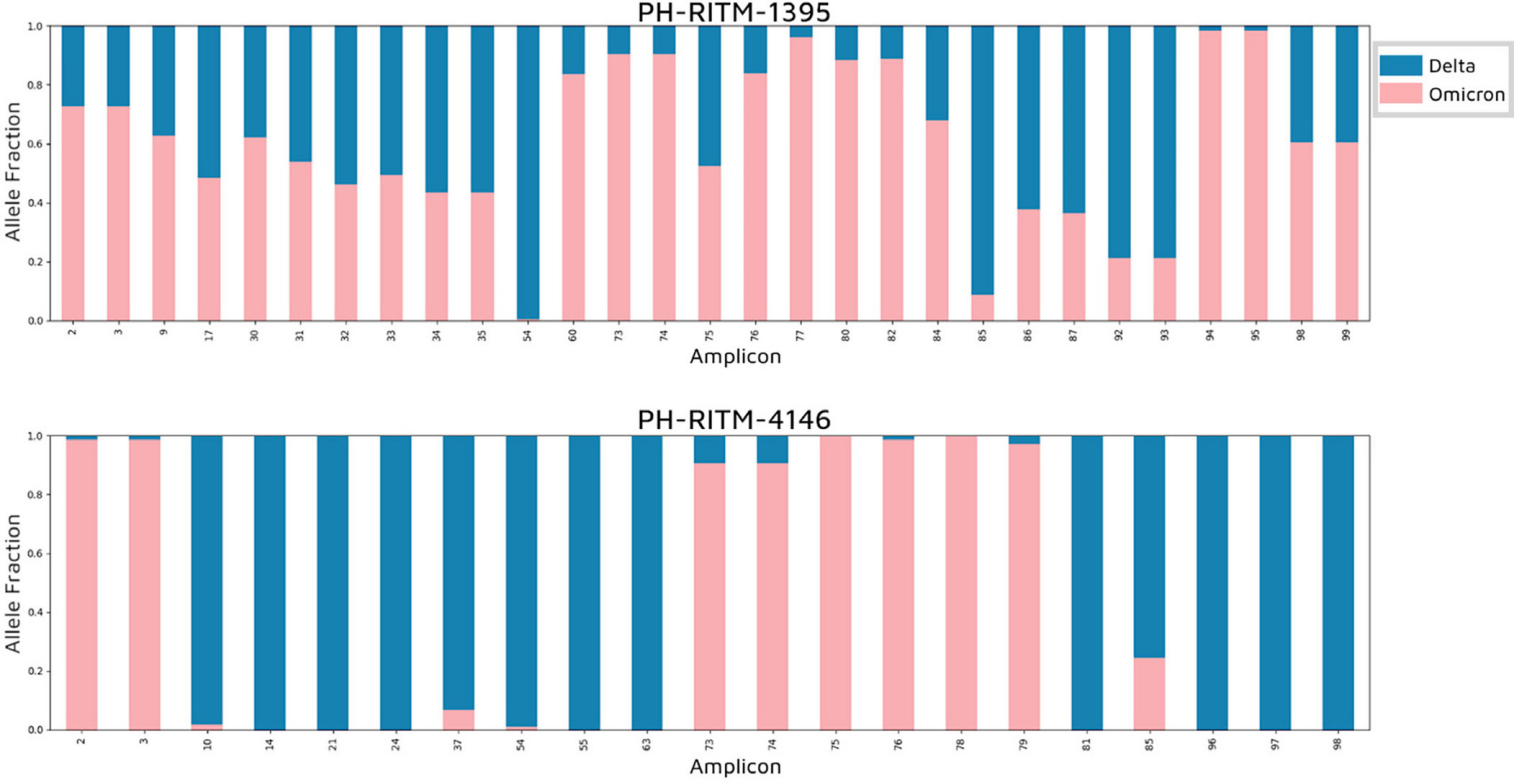
Alternative allele fraction per amplicon. The fraction of reads per amplicon having Delta (blue) and Omicron (pink) lineage-defining mutations was illustrated.

We then determined the AAF for each amplicon. PH-RITM-1395 shows a mixture of the two VOCs for almost all amplicons, supporting the pattern of co-infection. In contrast, PH-RITM-4146 displayed minimal mutation mixtures within amplicons, with distinct genomic regions showing amplicons corresponding exclusively to either Delta or Omicron. This pattern supports the sample as a recombinant variant with the possibility of the parental Omicron and Delta variants still present (Fig. 5).

Both the AAF plots per lineage-defining mutation and per amplicon support the hypothesis that PH-RITM-1395 contains a mixture of reads from both Delta and Omicron variants within a single amplicon, suggesting co-infection. In contrast, the AAF plots for PH-RITM-4146 show a pattern more indicative of recombination, with distinct regions of amplicons associated with either Delta or Omicron, possibly pointing to breakpoints within the sample. We inferred the breakpoints in PH-RITM-4146, illustrated in Fig. 4, to be between the mutations G142D-V213G and N679K-P681R, indicating that the breakpoints are located in the *S* gene.

#### Amplicon sorting

To further demonstrate the presence of mixed reads belonging to Omicron and Delta within a single sample, we sorted the reads associated with each variant. Utilizing the samjdk tool in jvarkit [26] for amplicon sorting, we effectively separated reads from the BAM file by identifying the lineage-defining mutations present in each read. Results from running custom Python scripts for amplicon sorting in Table 2 showed the assigned lineages for sample PH-RITM-1395 as B.1.617.2 (Delta) and BA.2 (Omicron) for the Pangolin tool, while Nextclade assigned them as B.1.617.2 (Delta) and BA.2.3 (Omicron). These results are concordant with VirStrain. On the other hand, the sorted sequences of sample PH-RITM-4146 were assigned as AY.1 (Delta) and B.1.1.529 (Omicron) by Pangolin, while Nextclade classified them as B.1.617.2 (Delta) and B.1 (Omicron). The lineage assignment for the Delta-specific reads in PH-RITM-4146 aligns with the initial results from both Pangolin (AY.1) and Nextclade (B.1.617.2). These findings further support the presence of reads originating from both Delta and Omicron within the sample, also underscoring the value of employing multiple tools to achieve a more comprehensive and reliable lineage assignment in samples suspected of co-infection.

**Table 2.** Lineage assignment after Amplicon sorting

Sample	Nextclade	Pangolin	Scorpio	Note
**PH-RITM-1395**	B.1.617.2	B.1.617.2	Delta (B.1.617.2-like)	UshER placements: B.1.617.2 (1/1)
	BA.2	BA.2.3	Probable Omicron (unassigned)	UShER placements: BA.2 (1/1)
**PH-RITM-4146**	AY.1	B.1.617.2	Delta (B.1.617.2-like) +K417 N	UShER placements: AY.1 (2/4), AY.122 (1/4), B.1.617.2 (1/4)
	B.1.617.2	B.1	Probable Omicron (unassigned)	UShER placements: B.1.1.529 (2/2); Scorpio found insufficient support to assign a specific lineage

### Confirmation of recombinant variant in PH-RITM-4146

Due to the allele fraction pattern observed in PH-RITM-4146, we hypothesized that the sample represents a recombinant and further analysed it using the sc2rf tool [37]. Sc2rf is a command-line program specifically designed for detecting potential SARS-CoV-2 recombinants by identifying regions in the genome where abrupt changes in lineage-defining mutations occur. The sc2rf analysis identified four potential breakpoint regions (between positions 22115–22200, 22882–22917, 23040–23055 and 23604–23854) in PH-RITM-4146 and suggested a recombinant between Delta (21J) and Omicron (BA.2 or 21L) (Fig. S2).

Meanwhile, from the allele fraction pattern shown in Fig. 4, we inferred possible breakpoint regions between the mutations G142D-V213G and N679K-P681R in the *S* gene. Mutations in the inferred breakpoint regions in the allele fraction plot correspond closely with the mutations also found at the breakpoint intervals detected by sc2rf: V213G (T22200G) and P681R (C23604G). Therefore, the breakpoints inferred from the allele fraction plot coincide with the breakpoints identified in sc2rf. In addition, mutation N679K (T23599G) in the inferred breakpoints in the allele fraction plot is also positioned close to a putative breakpoint region detected by sc2rf.

The consistent detection of breakpoints across multiple runs of the sc2rf tool, as well as the alignment of these breakpoints with lineage-specific mutations, supports the reliability of the recombination signal. Additionally, the location of the breakpoints corresponds to genomic regions where recombination events have been previously reported in SARS-CoV-2, further supporting the validity of the identification. According to Pipek *et al.* [6], recombination breakpoint hotspots were found to be at positions 22578–23202, 23525–23854 and 24130–24503 in the *S* gene.

To strengthen the evidence for recombination, we also examined the presence of lineage-defining mutations within the putative breakpoint region (22882–22992) and identified three reads out of 160 reads that carried mutations characteristic of both Delta and Omicron lineages (Fig. S3). Specifically, these reads contained the mutations T22882G (Omicron), T22917G (Delta) and G22992A (Omicron) within the same read segment, providing read-level evidence consistent with a recombinant genome.

These results further support the Freyja analysis, which detected recombinant lineages grouped in ‘Others’ with an average abundance of 12%. Specifically, Freyja identified signals consistent with recombination between Delta and Omicron, corresponding to the detection of XD, a known Delta–Omicron recombinant. The consistent identification of recombinant signals by both Freyja and sc2rf makes it unlikely that the ‘other’ variants detected by Freyja are due to misclassification.

### Pipeline development, validation on simulated mixtures, publicly available co-infection sequences, and retrospective application to routine surveillance samples

We developed a pipeline compiling the steps used to analyse these two flagged samples (Fig. 1). The pipeline is designed as a general tool for analysing sequences generated from routine SARS-CoV-2 genomic surveillance. It integrates community-standard tools to deliver comprehensive results, helping users to assess lineage discrepancies and identify potential co-infections or recombinant cases. The pipeline generates a summary report as an output, which includes the results from lineage assignment, flags for nucleotide mixtures and the alternative allele fraction plots.

To validate the pipeline’s applicability in detecting co-infections, we ran Katmon on Illumina simulated reads of randomized lineage combinations at varying proportions of major and minor allele (50 : 50, 80 : 20, 90 : 10, 95 : 5 and 98 : 2). Our results show that the pipeline can detect co-infections with minor alleles as low as 2% using Illumina reads by adjusting the bammix threshold for identifying nucleotide mixtures. Similarly, analysis of the ONT simulated Delta–Omicron co-infection demonstrated that the pipeline can reliably detect minor lineage proportions as low as 2%, even with a sequencing error percentage of up to 20%, as simulated using Badread [34]. Despite the inherently higher error profile of ONT data compared to Illumina, the pipeline maintained accurate detection of simulated co-infections across all tested error models (nanopore2020 and nanopore2023). Furthermore, semi-synthetic Delta–Omicron generated from pure ONT reads confirmed these findings, demonstrating consistent detection of co-infection across both simulated and semi-synthetic datasets (Figs S4F and S5).

To evaluate the performance of the pipeline on a panel of real-world data, we analysed publicly available sequences previously reported as co-infection cases [9,12,36]. These datasets included sequences generated using both Illumina and ONT platforms, allowing cross-validation of the pipeline across the two sequencing technologies. The full results of the simulated datasets and the confirmed co-infection samples are available in the repository (https://github.com/lanadelrea/simKatmon/tree/main/results).

Results from the Katmon pipeline were consistent with the findings reported in the aforementioned studies (Table S1, Figs S6-S8). For instance, sample SRR18272230 was previously described by Bolze *et al.* [*9*^9^] as a Delta–Omicron co-infection exhibiting signs of recombination. Katmon similarly detected the co-infection between the parental lineages Delta and Omicron, along with evidence of the recombinant lineage XS, a known Delta–Omicron recombinant. The pipeline also accurately identified the novel recombinant lineages XM and XS described by Perez-Florido *et al.* [12], which originated from parental lineages Omicron BA.2/BA.1.1 (XM) and Delta AY.98/Omicron BA.1.17 (XS). In addition to Delta/Omicron cases, Katmon successfully identified other reported co-infections, such as the Zeta (P.2) and Gamma (P.1) co-infection described by Dezordi *et al.*[36]. Overall, these results highlight the pipeline’s robustness in detecting diverse lineage combinations across multiple sequencing platforms and datasets.

**Fig. 6.**
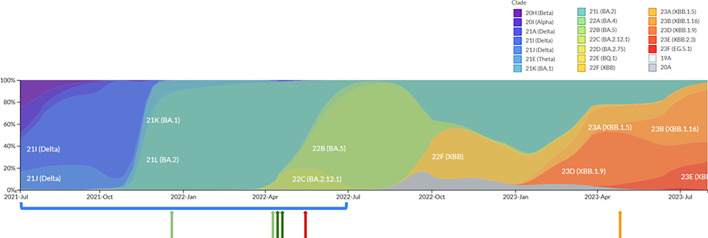
Lineage frequency plot of the Philippine SARS-CoV-2 sequences. The frequency plot illustrates lineage assignment, coloured by clade, for all publicly available Philippine samples collected from July 2021 to July 2023. The red and orange arrows, representing PH-RITM-1395 and PH-RITM-4146, respectively, indicate the samples initially investigated for co-infection and are the basis of the bioinformatics pipeline development. Out of 1,078 archived samples analysed, from the period of July 2021 to July 2022 (blue bracket), the pipeline detected a total of five possible co-infection cases: the sample PH-RITM-1395 (red arrow), two cases of Delta and Omicron co-infection (light green arrows) and two cases of Beta and Omicron co-infection (dark green arrows).

The subsequent application of the Katmon pipeline to archived samples further identified four potential co-infections, as shown in Fig. 6, with the earliest case dating back to December 2021. This brings the total number of co-infection cases in the Philippines to 5 out of 1,078 samples processed between July 2021 and July 2022. Among these, three cases involved co-infection with Delta and Omicron, while two involved Beta and Omicron. Hence, the prevalence of Delta and Omicron co-infections was 0.27%, and Beta and Omicron co-infections had a prevalence of 0.19%.

Notably, the Delta–Omicron co-infected samples originated from different batches and regions, with the first one collected from the National Capital Region (NCR) in December 2021 and the other one from Zamboanga (Region IX) in April 2022. Both collection dates predate the sample PH-RITM-1395 investigated in detail above, which was also from Zamboanga (Region IX). Meanwhile, the Beta-Omicron co-infected samples were collected within three days of each other in April 2022, from both the same batch and region (NCR).

## Discussion

In this study, we identified two potential cases of co-infection between SARS-CoV-2 variants Delta and Omicron through routine genomic surveillance during the COVID-19 pandemic in the Philippines. The two samples, PH-RITM-1395 and PH-RITM-4146, exhibited discordant lineage assignments and an unusually high number of private mutations flagged by Nextclade, prompting further investigation for possible contamination, co-infection or recombination. Elevated private mutation scores indicate the presence of additional mutations beyond those expected for a given lineage. While these scores alone are not specific enough to confirm contamination, co-infection or recombination, they can serve as an early warning signal that warrants closer examination of sequencing data [21]. After re-extracting and resequencing the samples to rule out cross-contamination, we developed and implemented a custom bioinformatics pipeline (*Katmon*). At the time of genomic surveillance, existing processes for detecting co-infections were limited, so we employed additional bioinformatics tools for a more stringent analysis of nucleotide mixtures. This thorough investigation enabled us to rule out cross-contamination and distinguish between cases of co-infection and the presence of two distinct variants and recombinants within a sample. Results from the simulated data demonstrate that the developed pipeline can detect co-infections with minor variants as low as 2% and can even identify co-infections of lineages belonging to the same clade. Applying the refined pipeline to archived samples, we uncovered four additional cases, including Delta–Omicron and Beta–Omicron co-infections. Our findings demonstrate that the persistence and co-circulation of different variants did result in co-infections, presenting the opportunity for recombination and the emergence of recombinant lineages.

Cases of co-infection between Omicron and Delta have been found in many countries, namely Australia, Argentina, Brazil, Equatorial Guinea, France, Norway, Singapore, Spain, South Africa and the USA [6,10,12, 21, 22, 36, 38]. Most of these, however, were from cases during the lineage replacement of Delta by Omicron from late 2021 to early 2022. In contrast, our first case, PH-RITM-1395, was detected in May 2022, a point in time in the Philippines when Omicron had become dominant, and Delta had reportedly only two cases during the said month. However, it is possible that the patient had a chronic infection that started earlier [41]. On the other hand, sample PH-RITM-4146 was collected in April 2023, a period when Delta lineages were no longer being reported through national genomic surveillance. The latest Delta Philippine sequence available on GISAID was collected in January 2023 from Davao and submitted by the Philippine Genome Center (PGC). Globally, only a limited number of Delta sequences (*n*=50) were reported in 2023 according to sequence data from GISAID [42]. While these may include potential date misannotations, we cannot fully exclude the possibility of low-level cryptic Delta circulation or under-detection due to limited sequencing coverage. Unfortunately, at this time, we have been unable to confirm whether these are collection date misannotations during submission.

Sample collection in this study was performed at a single point in time, prior to the development of our pipeline to automatically flag anomalous reads, so we were unable to observe any possible changes in relative abundance in the two variants over time. Previous studies have shown that longitudinal sampling is critical for understanding the dynamics of co-infection. For example, Pedro *et al.*[41] documented a case in which a patient initially presented with the absolute (100%) 20A lineage, followed by a minor 20B variant frequency (3%) nine days later, which eventually became the dominant lineage (100%) after two months. Similarly, Samoilov *et al.* [19] reported different abundances of clades GH and GR in swabs collected from the same patient taken eight days apart. Other work has also shown that Delta and Omicron can outcompete each other depending on the infection stage. For instance, Combes *et al.* [21] observed equal proportions of Delta and Omicron on day 1 of sample collection, but by day 11, Delta had largely displaced Omicron, accounting for 96% of lineage-defining mutations.

In light of these findings, we hypothesize that PH-RITM-4146 may represent either an early stage of dual infection, where Omicron had begun to co-infect alongside Delta, or a later stage in which Delta had already outcompeted Omicron. This dynamic can be understood in the context of clonal interference, a process wherein multiple viral lineages within the same host compete for replication and transmission. In such scenarios, only the lineage with a relative fitness advantage tends to dominate, while less fit variants decline in abundance [43,44]. In SARS-CoV-2, this competition between co-circulating lineages such as Delta and Omicron may determine which variant establishes dominance during co-infection, as observed in longitudinal studies where one lineage eventually supplanted the other [10,19, 21, 41].

Moreover, prolonged coexistence of competing lineages may increase the likelihood of recombination events, particularly in immunocompromised patients with persistent infections. Garcia *et al.* [10] further demonstrated that temporal sequencing of samples from a chronically infected and immunocompromised patient revealed competition between Delta AY.98 and Omicron BA.5, offering insights into the molecular mechanisms driving the emergence of recombinant lineages. This highlights the importance of temporal sampling in monitoring the evolution of viral populations and might be a point for consideration in improving genomic surveillance, particularly in patients with chronic COVID-19 infection [10,11, 19, 41, 45].

Although recombination is a well-documented mechanism in virus evolution, the molecular processes underlying the emergence of novel recombinant lineages remain poorly understood, particularly in the case of SARS-CoV-2, where genomic diversity is limited by its relatively short evolutionary history [6,10, 16]. Genomic surveillance has shown that co-infections, although relatively rare, occur often enough to provide opportunities for recombination and the emergence of new lineages [10,12, 39]. When genetically distinct strains co-exist within a patient, recombination can give rise to viral genomes with unique mutational landscapes [6,45, 46]. A study by Tremeaux *et al.* [45] reported that 24% of the co-infection cases investigated harboured recombinant viruses. Beyond the risk of new variants, certain co-infections have also been associated with more severe clinical outcomes and prolonged disease duration [19,41]. Detecting these cases in a timely manner is, therefore, critical for guiding diagnostics, patient care and clinical management.

Distinguishing between co-infection and recombinant variants requires careful interpretation of allele frequency patterns. Co-infections typically present with mixed alleles from different lineages distributed throughout the genome, whereas recombinant viruses show lineage-defining mutations localized to distinct genomic regions [9]. The allele fraction pattern for sample PH-RITM-1395 is consistent with co-infection signals; therefore, we identified the sample as having true dual infection.

In a recombinant genome, the consensus sequence reflects the parental contributions, with subsets of lineage-defining mutations from both strains arranged according to recombination breakpoints [6,16]. Its detection *in vivo* is challenging unless the parental strains are genetically distinct. When the genomes involved are identical or nearly identical, recombination events can be harder to trace [46]. Recombination is thought to occur frequently during co-infections; however, early recombination events within a host often remain undetectable in consensus sequences, as parental genomes dominate the sample and recombinant genomes appear only at low frequencies. Detecting such intra-host recombination requires analysis of raw sequencing reads, rather than consensus assemblies, to identify subtle shifts in allele fractions [6,46].

Our analysis of PH-RITM-4146 illustrates these challenges. One interpretation is that the sample represents a co-infection with Delta, Omicron, and a recombinant of the two, where all three genomes co-exist in varying proportions. Alternatively, it may capture a transient stage of intra-host recombination, in which only a fraction of reads contains recombinant signatures. This interpretation is supported by both Freyja and sc2rf analyses, which consistently detected signals of recombinant lineages, including XD, a known Delta–Omicron recombinant. The concordance between these independent methods reduces the likelihood that the observed ‘other variants’ in Freyja were artefacts of misclassification, instead suggesting genuine recombinant fragments within the viral population. The presence of these minor recombinant signals, averaging 12% abundance, implies that recombination may have occurred within the host, potentially during the replication of co-infecting Delta and Omicron variants. However, there is no record of the XD lineage circulating in the Philippines, suggesting that it did not spread further or was not detected by local genomic surveillance.

As sequencing technologies advance, especially with the increasing availability of high-quality long-read approaches, distinguishing recombinant genomes from co-infections will become more feasible. Long-read sequencing (e.g. PacBio or ONT platforms) has already demonstrated the ability to resolve complex viral populations and characterize co-infections and recombinants with greater accuracy [11,45]. These methods reduce contamination risk, mitigate artefacts associated with short-read approaches and take advantage of improved raw error rates in third-generation sequencing [45]. Integrating long-read sequencing into genomic surveillance would, therefore, provide a complementary approach to short-read data, facilitating more reliable detection of co-infections, recombinant strains and the mechanisms driving SARS-CoV-2 evolution.

Estimates of the prevalence of co-infections have been calculated in various studies worldwide. In Argentina, there was a 0.1% prevalence of Delta–Omicron co-infection over a year period from December 2021 to January 2022 [39]. Meanwhile, the first case of dual infection with other VOCs, namely Beta and Delta, was reported in Equatorial Guinea with an overall prevalence of co-infection in the country at 2.1% [38]. In France, a study that screened 3,237 samples for VOC-specific SNPs observed 0.2% of samples co-infected with Delta and Omicron and detected a recombination event between the two variants [21]. Another study in France performed on 15,253 samples determined the co-infection with Delta/Omicron (BA.1) and BA.1/BA.2 Omicron lineages, which were estimated to have a lower prevalence of 0.18% and 0.26%, respectively, for the period December 2021 to February 2022 [22]; these percentages are similar to those we have calculated for the Philippines. In our dataset of 1,078 samples, the prevalence of Delta/Omicron co-infections was 0.27%, while Beta/Omicron co-infections were observed at 0.19%. Meanwhile, co-infection rates vary considerably for each country. For example, the reported rate of co-infection in the USA was 0.3–0.5% from January to September 2021 [40]. South Africa reported one of the highest rates of 0.92% due to the country also having the largest percentage of immunocompromised population [6]. A study by Pipek *et al.* [6] reported that the worldwide estimate of the co-infection rate is 0.35%. Overall, these findings indicate that while co-infections with SARS-CoV-2 variants have been documented across different regions, their prevalence and rates remain relatively low, suggesting that such cases are uncommon despite the concurrent circulation of multiple lineages. However, this is potentially due to the relatively small number of studies, the limited sensitivity of detection methods, undersampling, or the challenges in identifying mixed infections from sequencing data. Furthermore, although our prevalence estimates are comparable to those reported elsewhere, prevalence calculations are descriptive measures. These caveats suggest that our findings should be interpreted cautiously and highlight the importance of complementary validation through using probabilistic models describing the occurrence of rare events, such as co-infections [47,48].

Our initial investigation of the two cases prompted the development of our *Katmon* pipeline, which streamlines the detection of co-infection cases in routine sequencing efforts. The pipeline generates an HTML report summarizing lineage assignment, flagged positions with nucleotide mixtures, alternative allele fraction patterns and results of the amplicon sorted reads, all derived from a combination of community-standard tools and custom scripts. This report will serve as a valuable resource for researchers and public health officials in monitoring and analysing co-infection cases. Our pipeline can be employed in (low- and middle-income countries) LMIC laboratories, such as in the Philippines, as it utilizes only open-source and publicly available tools without the need for more computationally intensive infrastructure.

We validated the pipeline using confirmed co-infection cases from France, Brazil, Australia, Spain and Norway [9,12,36], and the results were consistent with the findings of these studies. For example, our pipeline successfully detected the reported co-infections of the P.1 (Zeta) lineage with Gamma variants in Brazil. It also identified multiple Omicron variants within a single sample from Spain [12] and Delta–Omicron co-infections in samples from France, Brazil and Australia [9,11, 36]. Notably, most of these studies relied on Illumina sequencing technology, which is widely used for WGS, highlighting that our pipeline is applicable across both Illumina and ONT datasets.

In our retrospective analysis of the Philippine dataset, the additional co-infections detected by the pipeline highlight both the temporal and geographic spread of these events in the country. The Delta–Omicron co-infections detected using the pipeline were collected from distinct regions and time points: one from NCR in December 2021 and another from the Zamboanga region in April 2022. The NCR case was collected earlier than the PGC-reported case [17] from the same region, suggesting that co-infections were already occurring in the Philippines as early as December 2021, coinciding with the rise of Omicron while Delta was still circulating. Meanwhile, the Zamboanga case showed similarities to PH-RITM-1395, sampled a month later in the same region, indicating that recombinants may have persisted locally. Interestingly, the two Beta-Omicron co-infections were collected within days of each other in April 2022 and processed in the same sequencing batch, raising questions about possible epidemiological links that we cannot confirm due to limited access to patient data. Factors such as vaccination status, disease severity and clinical outcomes could provide further context but remain unavailable. Finally, the concentration of co-infection cases in NCR and Zamboanga may partly reflect uneven sampling within regions of the Philippines, underscoring the importance of more geographically representative genomic surveillance in the country.

There are a number of limitations to our study and the developed pipeline. First, the pipeline requires different input formats for the various tools, and the way files are processed may differ across laboratories. We specifically use the ARTIC pipeline to generate consensus FASTA and BAM files. Second, the pipeline is primarily designed to detect putative co-infections; cases such as the second sample, which may be undergoing intra-host recombination, require closer manual inspection with additional tools. Third, the limited metadata available for the samples, such as vaccination status, disease severity or patient outcome, makes it challenging to establish epidemiological links. Finally, due to resource constraints, we were unable to perform longitudinal sampling, which would be valuable in future work, particularly when investigating the potential emergence of recombinant variants.

Despite these limitations, our findings provide evidence of multiple co-infection events in the Philippines during periods of variant co-circulation. These observations underscore the importance of continued genomic surveillance and highlight the potential of bioinformatics pipelines to detect complex infections that may otherwise go unnoticed. Retrospective analyses could also uncover previously unrecognized co-infections, while prospective detection through routine integration of the Katmon pipeline could enhance real-time genomic surveillance. Such integration may enable the early identification of precursors to recombination, which can drive the emergence of new variants and influence patient outcomes or outbreak trajectories.

## Supplementary material

10.1099/mgen.0.001604 Uncited Supplementary Material 1.

## Abbreviations

AAF: alternative allele fraction
COVID-19: coronavirus disease 2019
EB: epidemiology bureau
GISAID: global initiative on sharing all influenza data
NCR: national capital region
NEC: negative extraction control
ONT: Oxford nanopore technology
PGC: Philippine genome center
qPCR: quantitative PCR
RITM: research institute for tropical medicine
RT-qPCR: reverse transcription qualitative real time PCR
SARS-CoV-2: severe acute respiratory syndrome 2
SNVs: single-nucleotide variants
VOC: variants of concern
WGS: whole-genome sequencing
