# Supplementary material for: Integration of bioinformatic tools for the detection of SARS-CoV-2 co-infection cases
Source: Microb Genom. 2026 Jan 29;12(1):001604. doi: 10.1099/mgen.0.001604 (PMC12856159; doi:10.1099/mgen.0.001604)
Supplement: Uncited Supplementary Material 1. [file mgen-12-01604-s001.pdf]

Supplementary Figure 1. Nextclade flagged private mutations of the sample. Nextclade flags for the samples PH-RITM-1395 and PH-RITM-4146 not passing the QC for private mutations, listing both labeled and unlabeled private mutations.

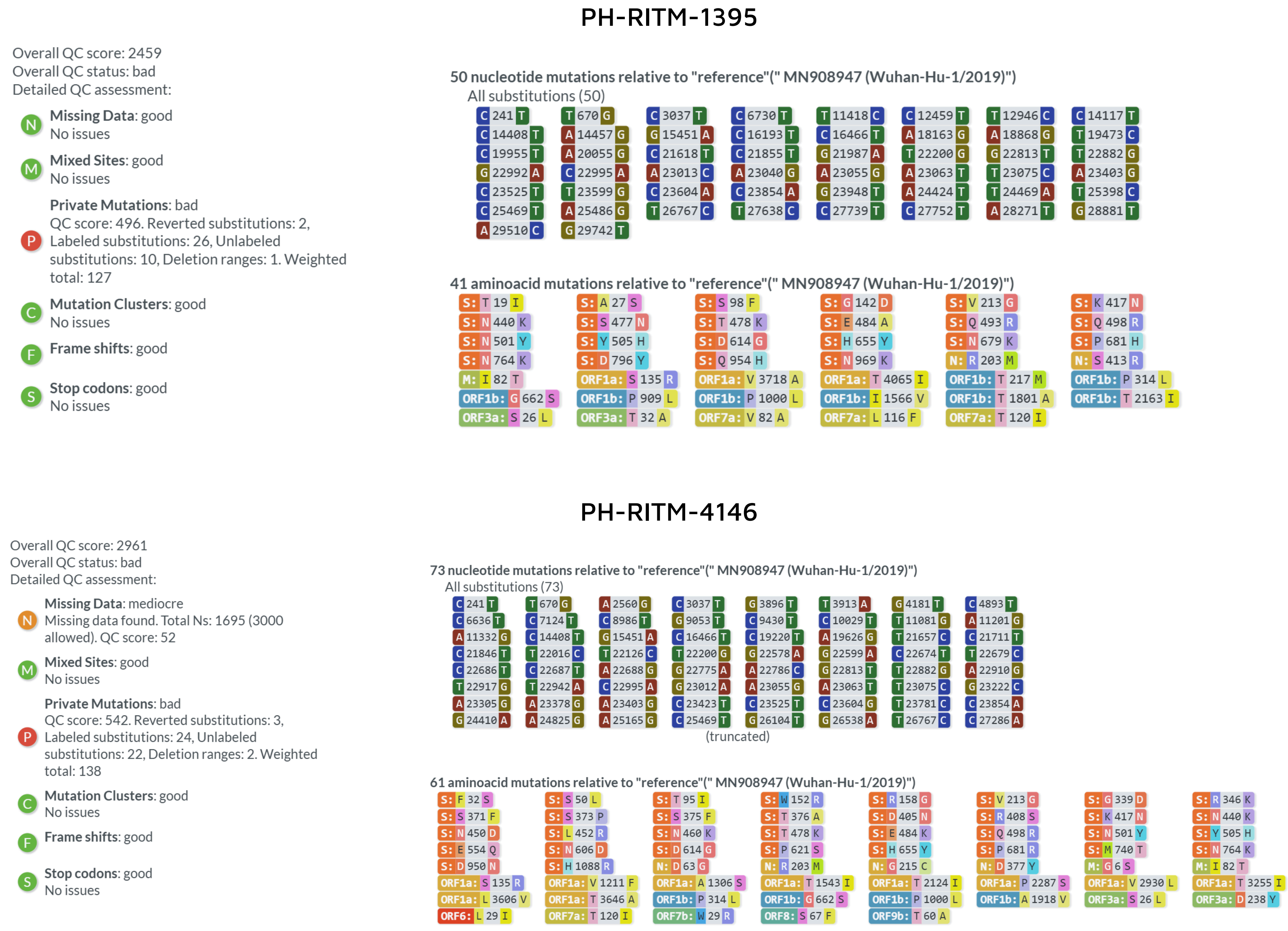

Supplementary Figure 2. Screenshot of sc2rf results showing that the sample PH-RITM-4146 is a possible recombinant of Delta (21J) and Omicron (BA.2/21L), having four breakpoints indicated by pink vertical lines.

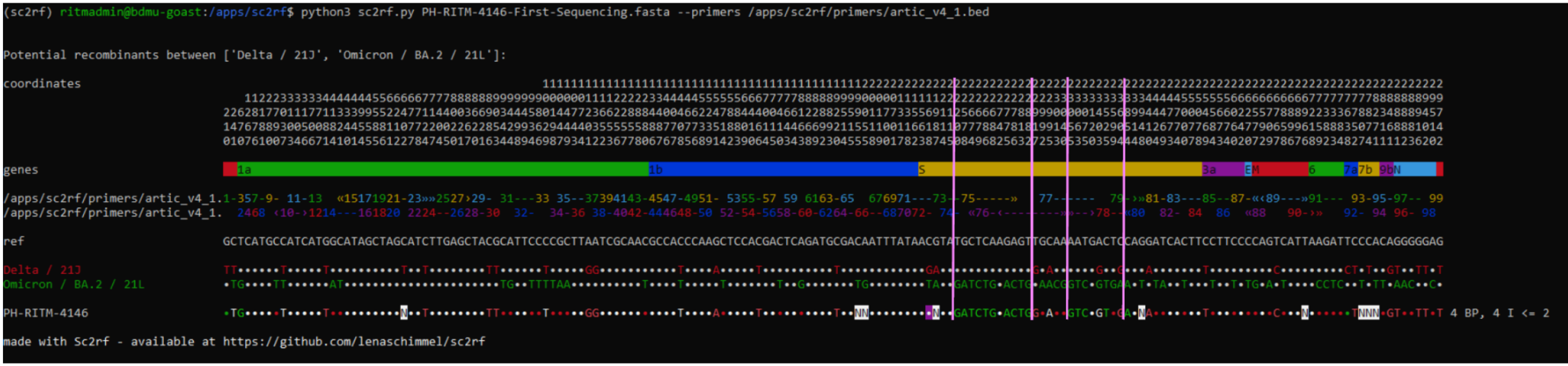

PH-RITM-4146 First Sequencing

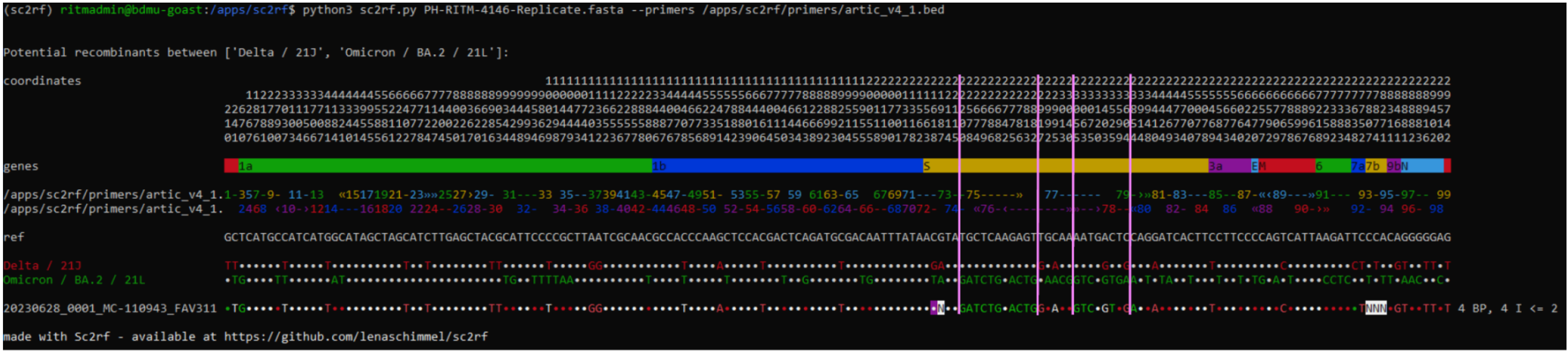

PH-RITM-4146 Replicate

Supplementary Figure 3. Three reads (highlighted in orange, green, and purple) in a putative breakpoint region contain both Delta and Omicron lineage-defining mutations as viewed in IGV. Specifically, the mutations are T22882G (Omicron), T22917G (Delta), and G22992A (Omicron) and are boxed in red.

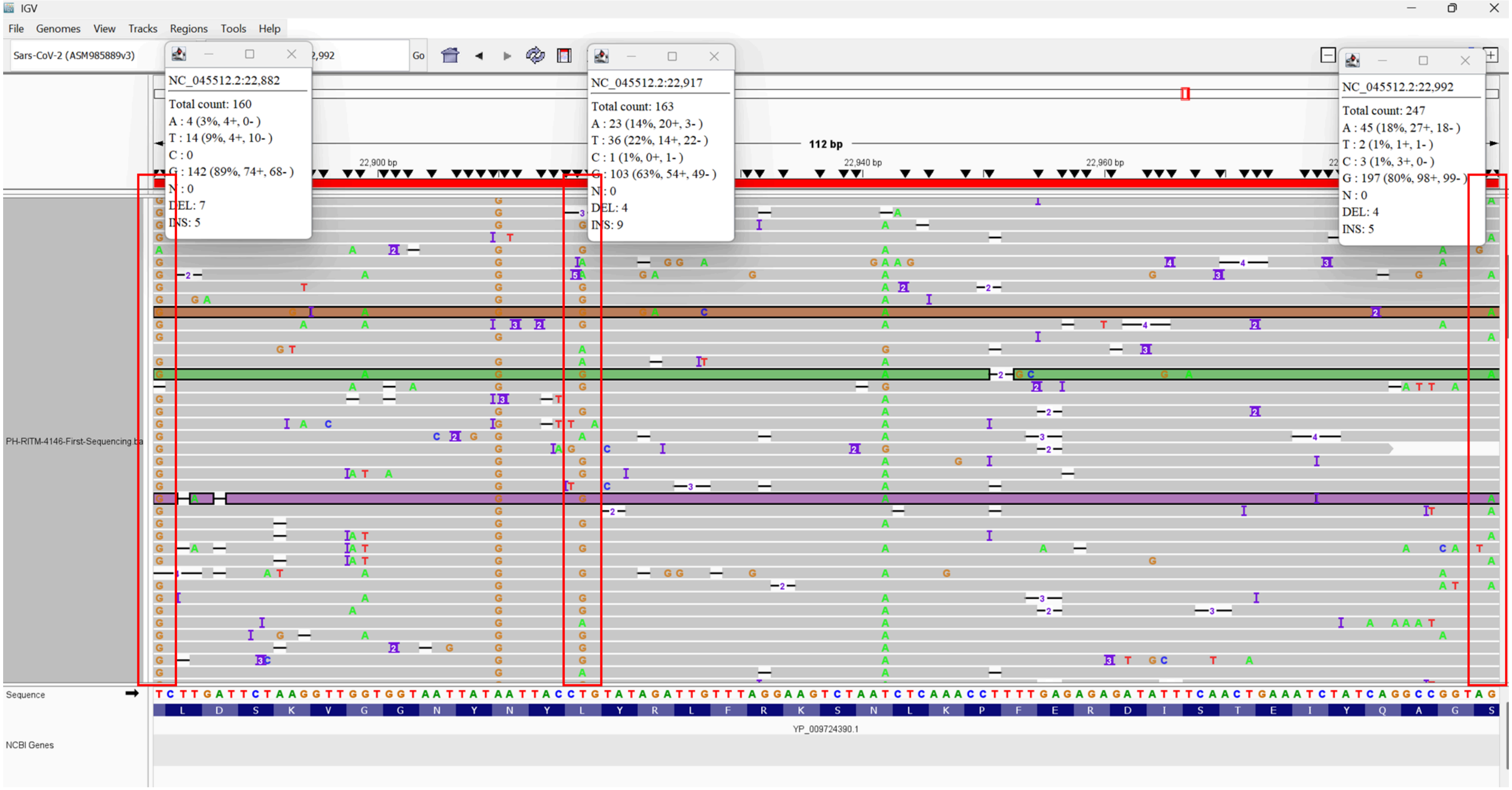

Supplementary Figure 4. Lineage abundance results of the Illumina and ONT simulated reads, and semi-synthetic mixed reads. The Illumina reads were simulated using ww_simulations, ONT reads were simulated using Badread, and the semi-synthetic mixed FASTQ files were created using rasusa. (A) Illumina simulated co-infection of Delta AY.107 and Omicron BA.2.3. (B) Illumina simulated co-infection of Delta AY.132 and Delta AY.63. (C) Illumina simulated co-infection of Omicron BA.1.10 and Omicron BA.2.5. (D) ONT simulated co-infection using nanopore2020 error model; (E) ONT simulated co-infection using nanopore2023 error model. (F) Mixed reads from Delta (SRX26994353) and Omicron BA.2 (SRX27000564) raw ONT reads.

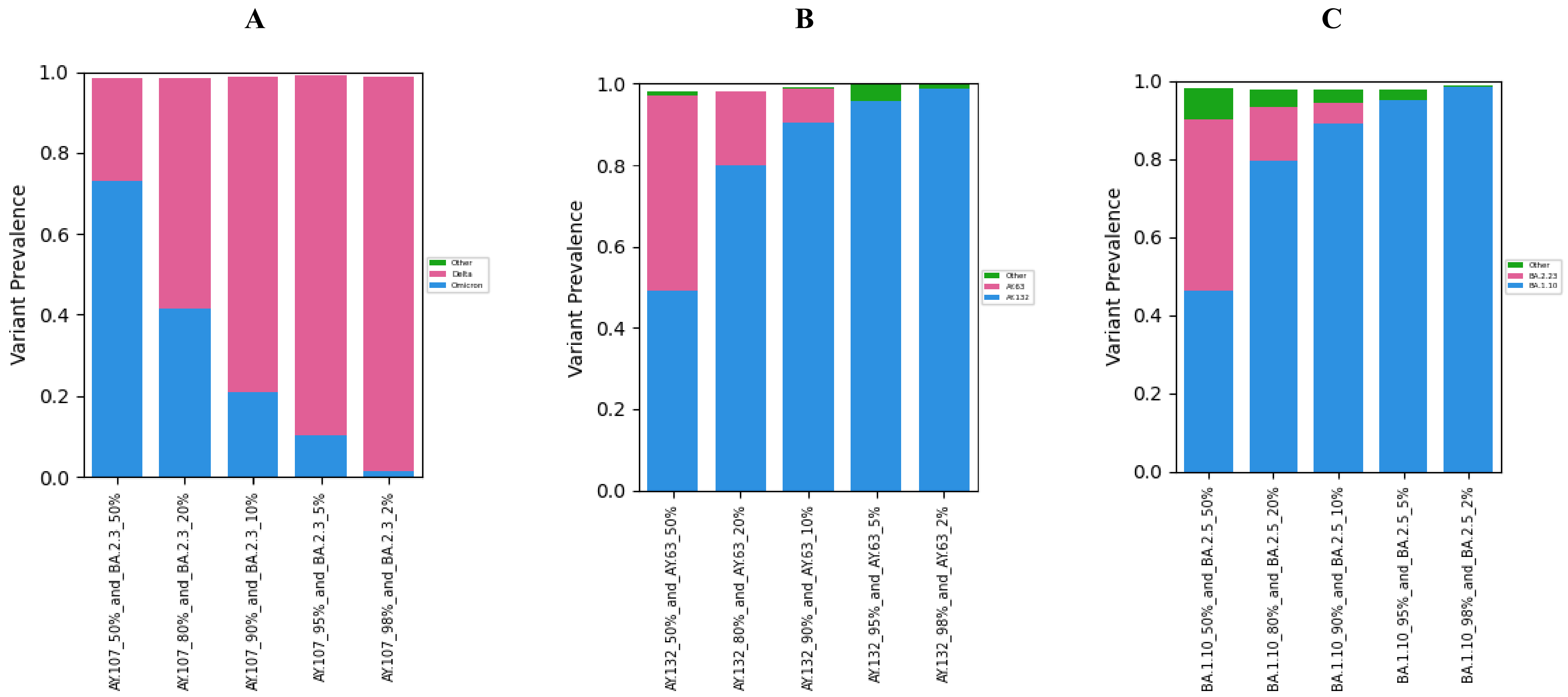

D

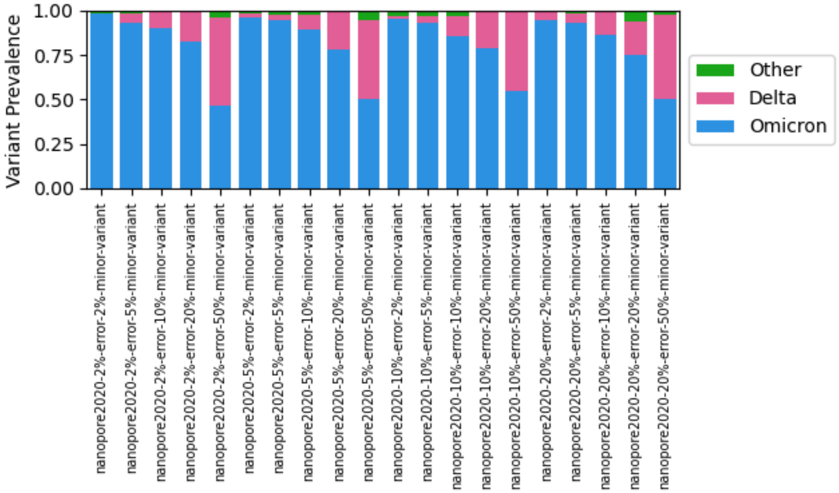

Variant Prevalence
1.00
0.75
0.50
0.25
0.00
Other
Delta
Omicron
nanopore2020-2%-error-2%-minor-variant
nanopore2020-2%-error-5%-minor-variant
nanopore2020-2%-error-10%-minor-variant
nanopore2020-2%-error-20%-minor-variant
nanopore2020-2%-error-50%-minor-variant
nanopore2020-5%-error-2%-minor-variant
nanopore2020-5%-error-5%-minor-variant
nanopore2020-5%-error-10%-minor-variant
nanopore2020-5%-error-20%-minor-variant
nanopore2020-5%-error-50%-minor-variant
nanopore2020-10%-error-2%-minor-variant
nanopore2020-10%-error-5%-minor-variant
nanopore2020-10%-error-10%-minor-variant
nanopore2020-10%-error-20%-minor-variant
nanopore2020-10%-error-50%-minor-variant
nanopore2020-20%-error-2%-minor-variant
nanopore2020-20%-error-5%-minor-variant
nanopore2020-20%-error-10%-minor-variant
nanopore2020-20%-error-20%-minor-variant
nanopore2020-20%-error-50%-minor-variant

E

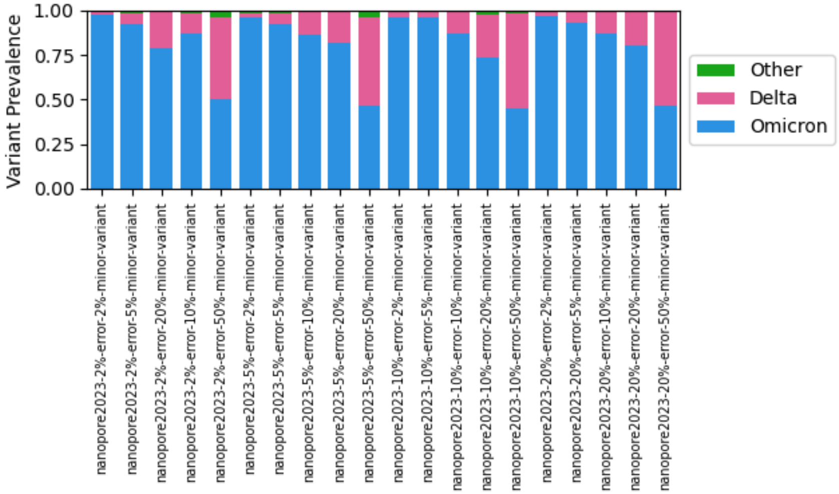

Variant Prevalence
1.00
0.75
0.50
0.25
0.00
Other
Delta
Omicron
nanopore2023-2%-error-2%-minor-variant
nanopore2023-2%-error-5%-minor-variant
nanopore2023-2%-error-20%-minor-variant
nanopore2023-2%-error-10%-minor-variant
nanopore2023-2%-error-50%-minor-variant
nanopore2023-5%-error-2%-minor-variant
nanopore2023-5%-error-5%-minor-variant
nanopore2023-5%-error-10%-minor-variant
nanopore2023-5%-error-20%-minor-variant
nanopore2023-5%-error-50%-minor-variant
nanopore2023-10%-error-2%-minor-variant
nanopore2023-10%-error-5%-minor-variant
nanopore2023-10%-error-10%-minor-variant
nanopore2023-10%-error-20%-minor-variant
nanopore2023-10%-error-50%-minor-variant
nanopore2023-20%-error-2%-minor-variant
nanopore2023-20%-error-5%-minor-variant
nanopore2023-20%-error-10%-minor-variant
nanopore2023-20%-error-20%-minor-variant
nanopore2023-20%-error-50%-minor-variant

F

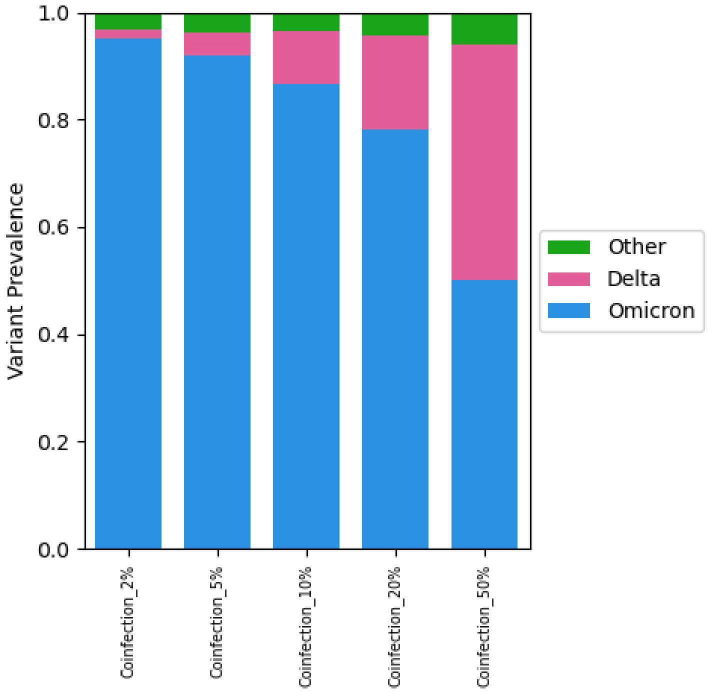

Variant Prevalence
1.0
0.8
0.6
0.4
0.2
0.0
Other
Delta
Omicron
Coinfection_2%
Coinfection_5%
Coinfection_10%
Coinfection_20%
Coinfection_50%

Supplementary Figure 5. Nucleotide mixtures of semi-synthetic files from raw ONT reads from Delta (SRX26994353) and Omicron BA.2 (SRX27000564): (A) 2% minor variant proportion; (B) 5% minor variant proportion; (C) 10% minor variant proportion; (D) 20% minor variant proportion; and (E) 50% minor variant proportion.

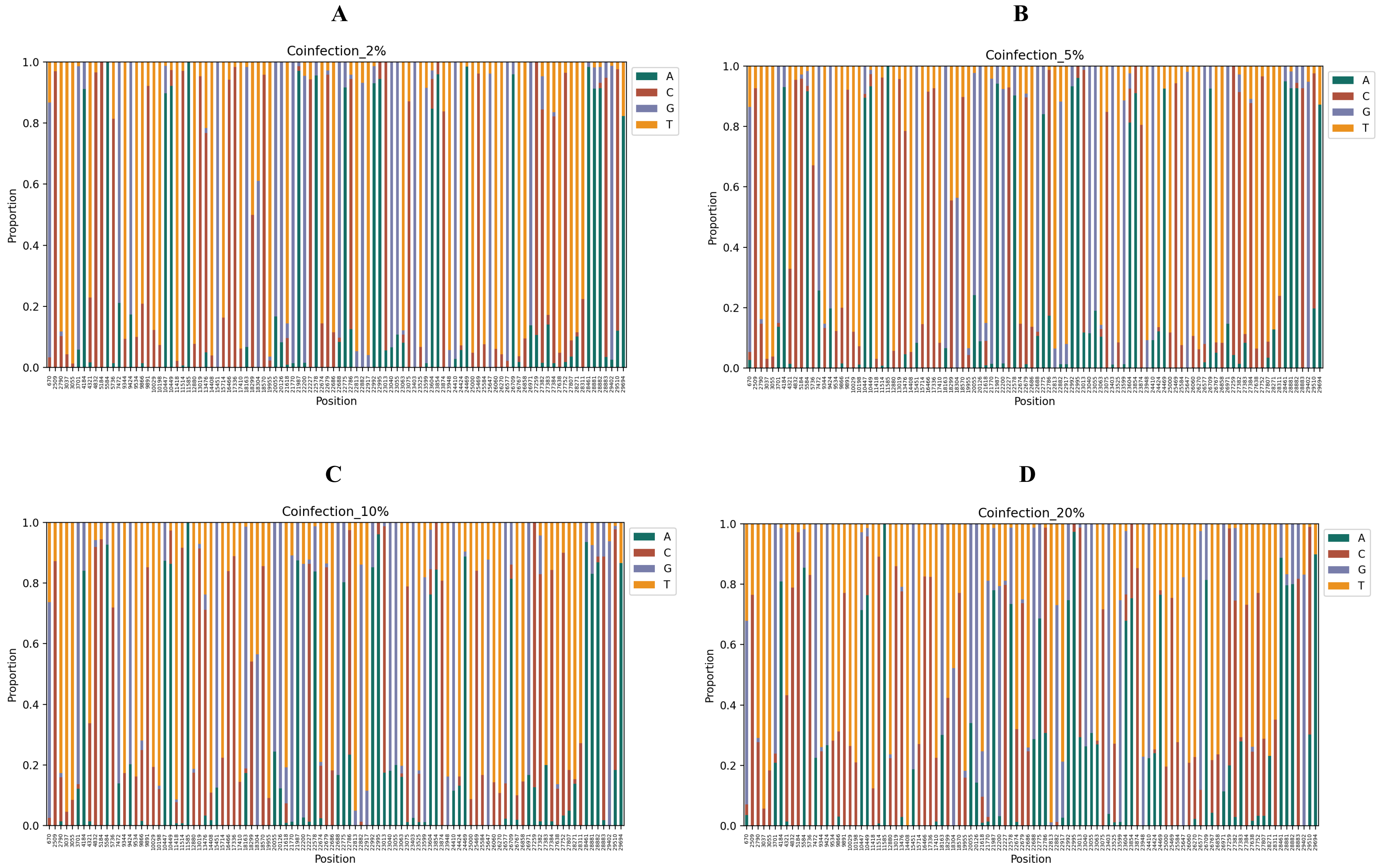

E

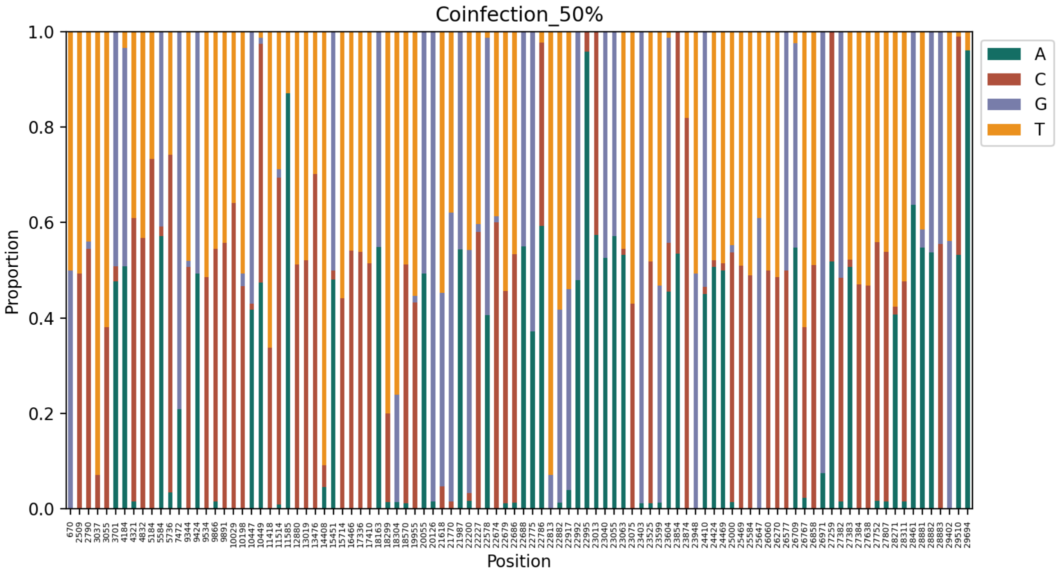

Supplementary Figure 6. Lineage abundance of confirmed and publicly available co-infection datasets: (A) Bolze et al. (2022); (B) Dezordi et al. (2022); (C) Garcia et al. (2024); (D) Perez-Florido et al. (2023); (E) Rockett et al. (2022) Illumina and (F) ONT samples.

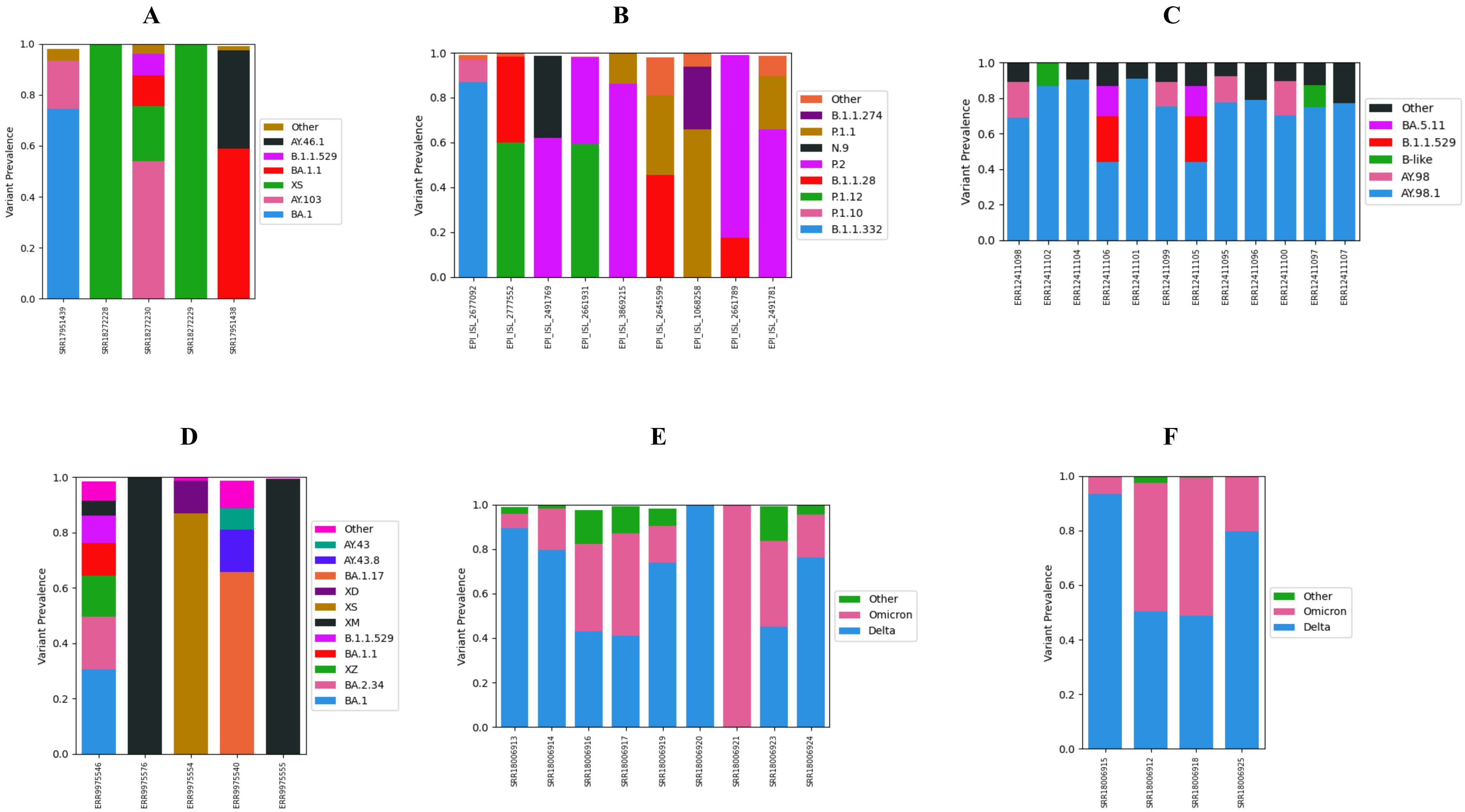

Supplementary Figure 7. Nucleotide mixtures per position of confirmed and publicly available co-infection samples. (A) Bolze et al. sample SRR17951438; (B) Dezordi et al. sample EPI_ISL_2491781; (C) Garcia et al. sample ERR12411105; (D) Perez-Florido et al. sample ERR9975540; (E) Rockett et al. Illumina sample SRR18006917 and (F) ONT sample SRR18006912.

A

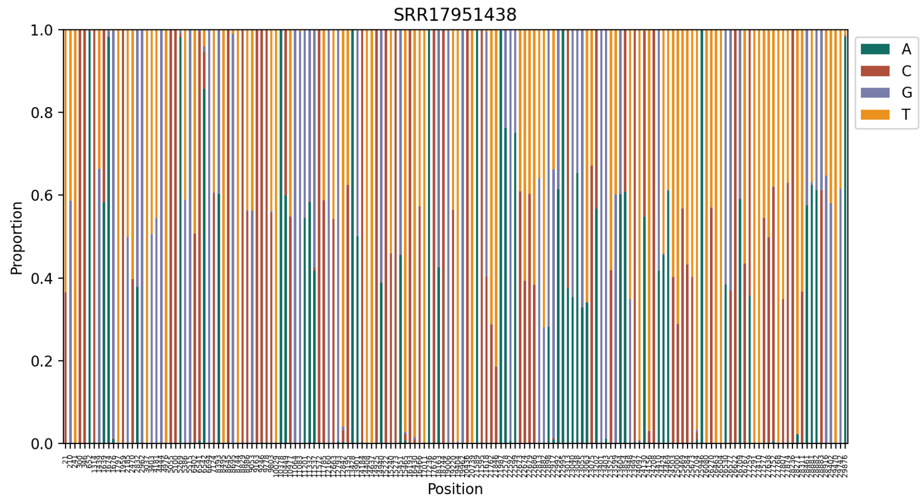

B

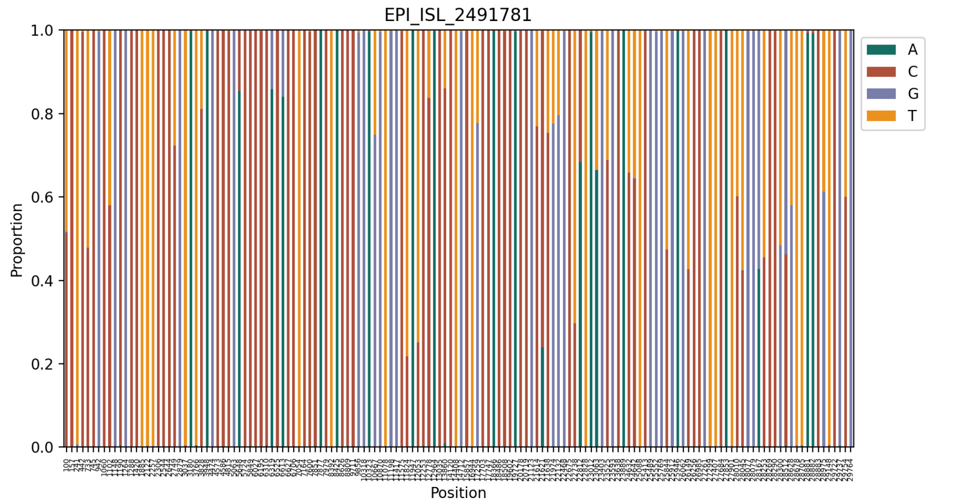

C

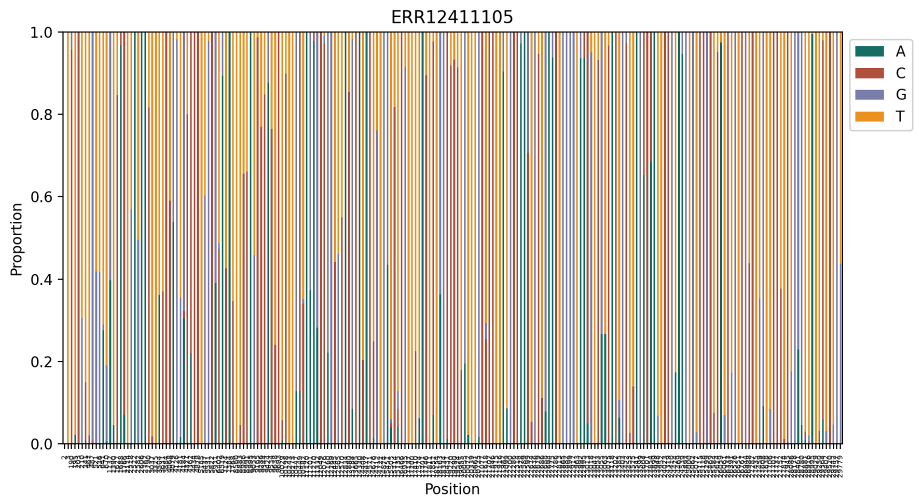

D

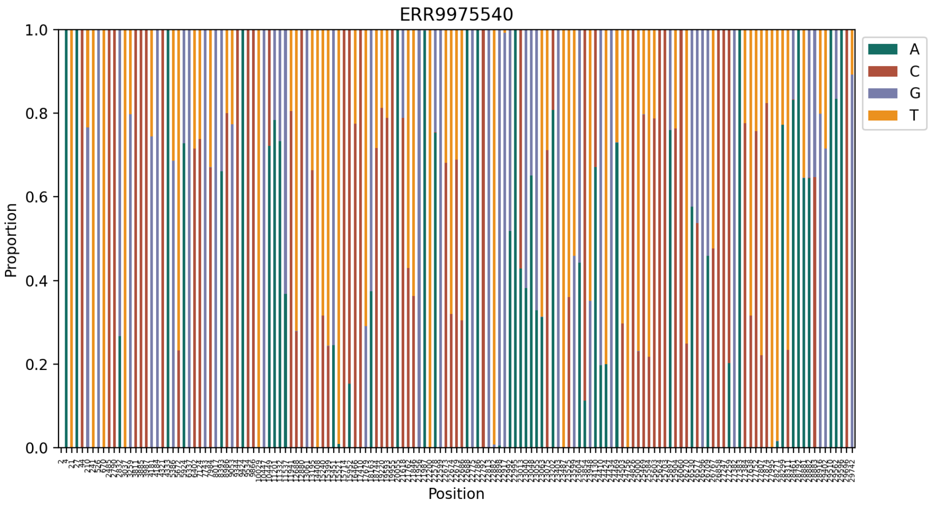

E

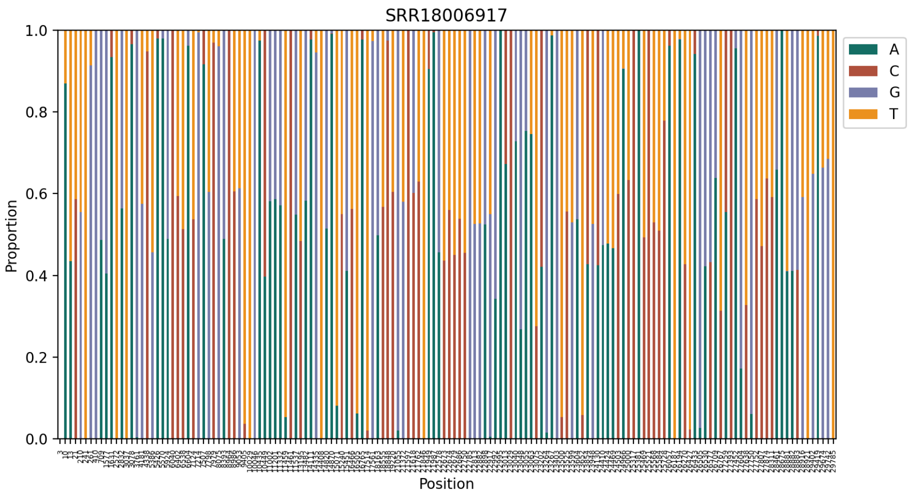
SRR18006917
Proportion
Position
A
C
G
T

F

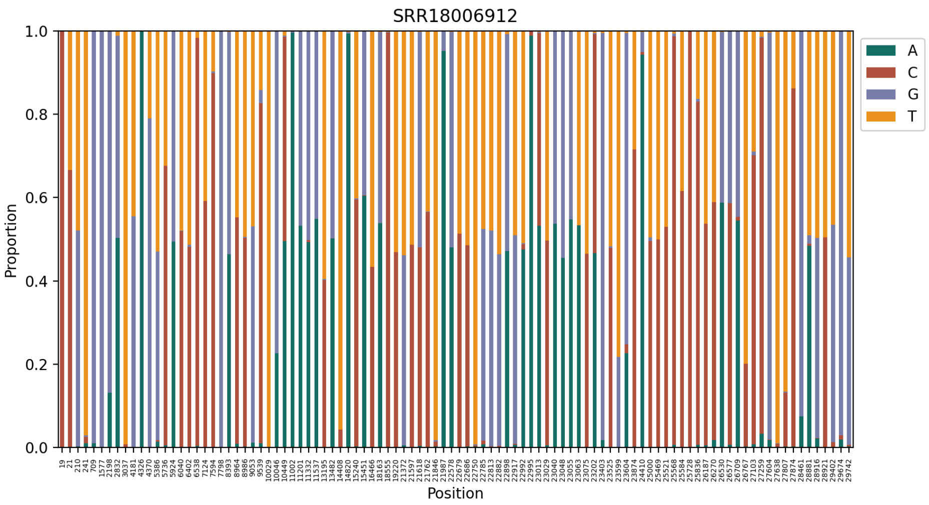
SRR18006912
Proportion
Position
A
C
G
T

Supplementary Figure 8. Allele fraction plot results per mutation and amplicon of the samples from confirmed and publicly available co-infections. (A) Bolze et al. sample SRR17951438; (B) Dezordi et al. sample EPI_ISL_2491781; (C) Garcia et al. sample ERR12411105; (D) Perez-Florido et al. sample ERR9975540; (E) Rockett et al. Illumina sample SRR18006917 and (F) ONT sample SRR18006912.

A

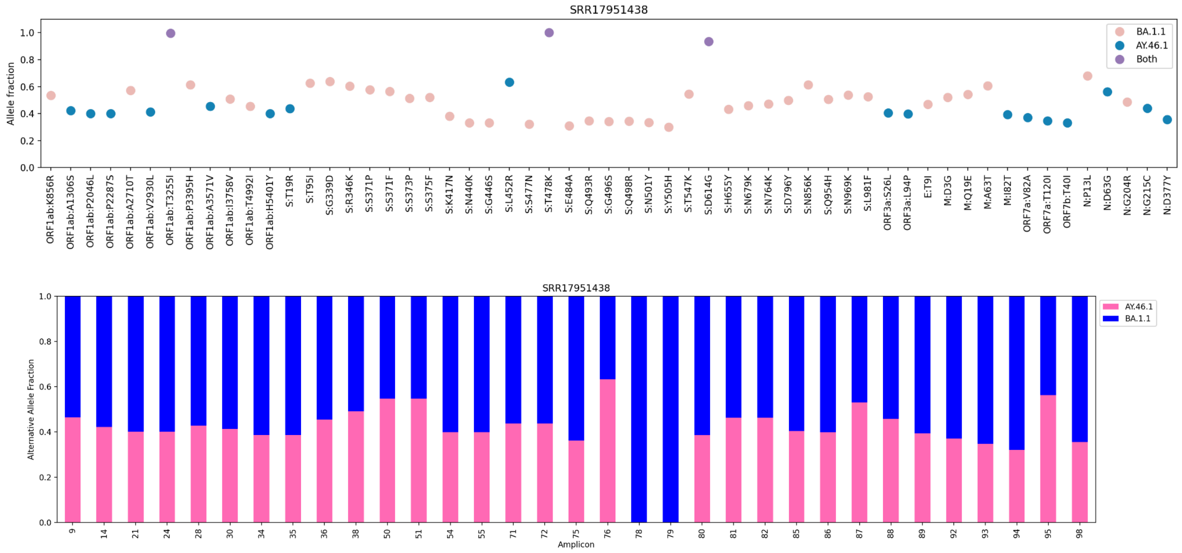

B

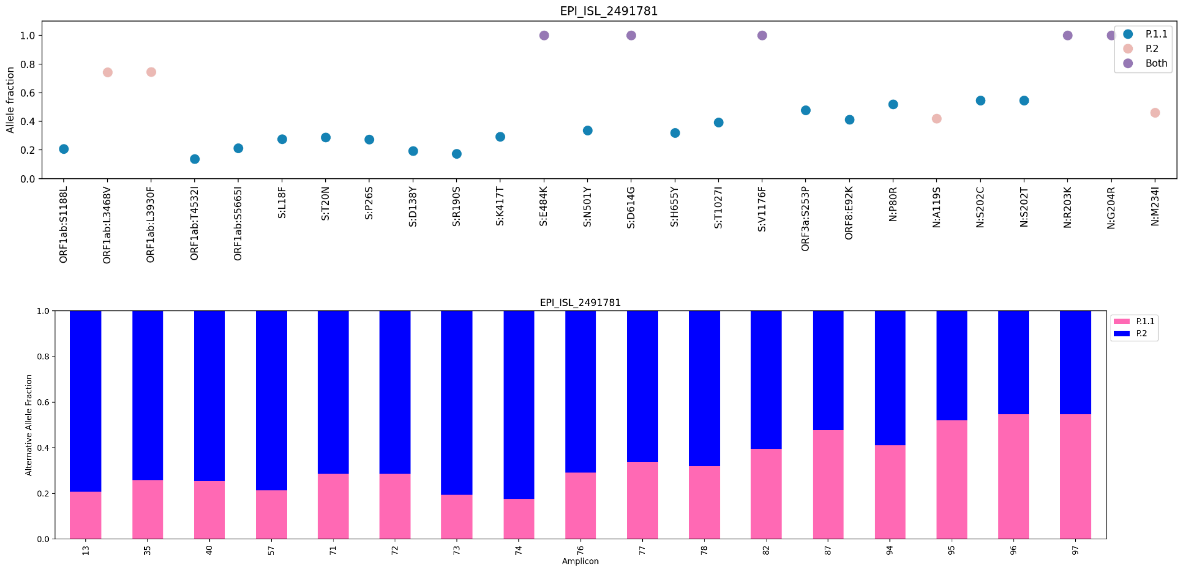

C

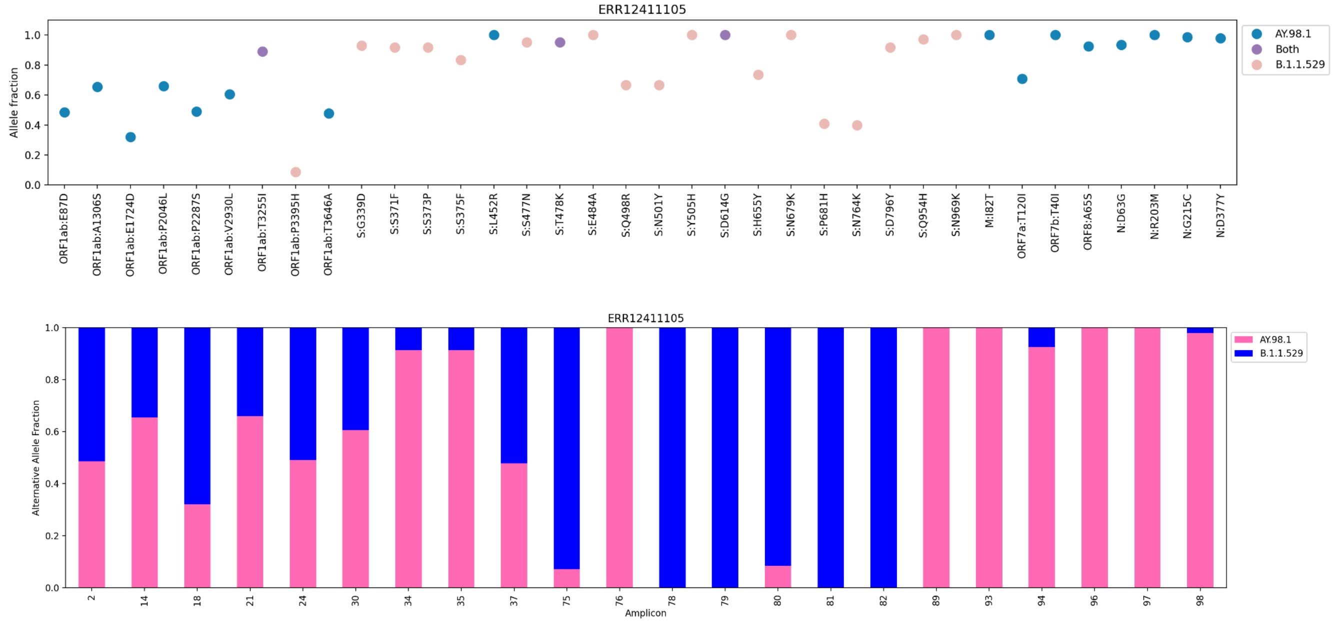
ERR12411105
Allele fraction
1.0
0.8
0.6
0.4
0.2
0.0
ORF1ab:E87D
ORF1ab:A1306S
ORF1ab:E1724D
ORF1ab:P2046L
ORF1ab:P2287S
ORF1ab:V2930L
ORF1ab:T3255I
ORF1ab:P3395H
ORF1ab:T3646A
S:G339D
S:S371F
S:S373P
S:S375F
S:L452R
S:S477N
S:T478K
S:E484A
S:Q498R
S:N501Y
S:Y505H
S:D614G
S:H655Y
S:N679K
S:P681H
S:N764K
S:D796Y
S:Q954H
S:N969K
M:I82T
ORF7a:T120I
ORF7b:T40I
ORF8:A65S
N:D63G
N:R203M
N:G215C
N:D377Y
AY.98.1
Both
B.1.1.529
ERR12411105
Alternative Allele Fraction
1.0
0.8
0.6
0.4
0.2
0.0
2
14
18
21
24
30
34
35
37
75
76
78
79
80
81
82
89
93
94
96
97
98
Amplicon
AY.98.1
B.1.1.529

D

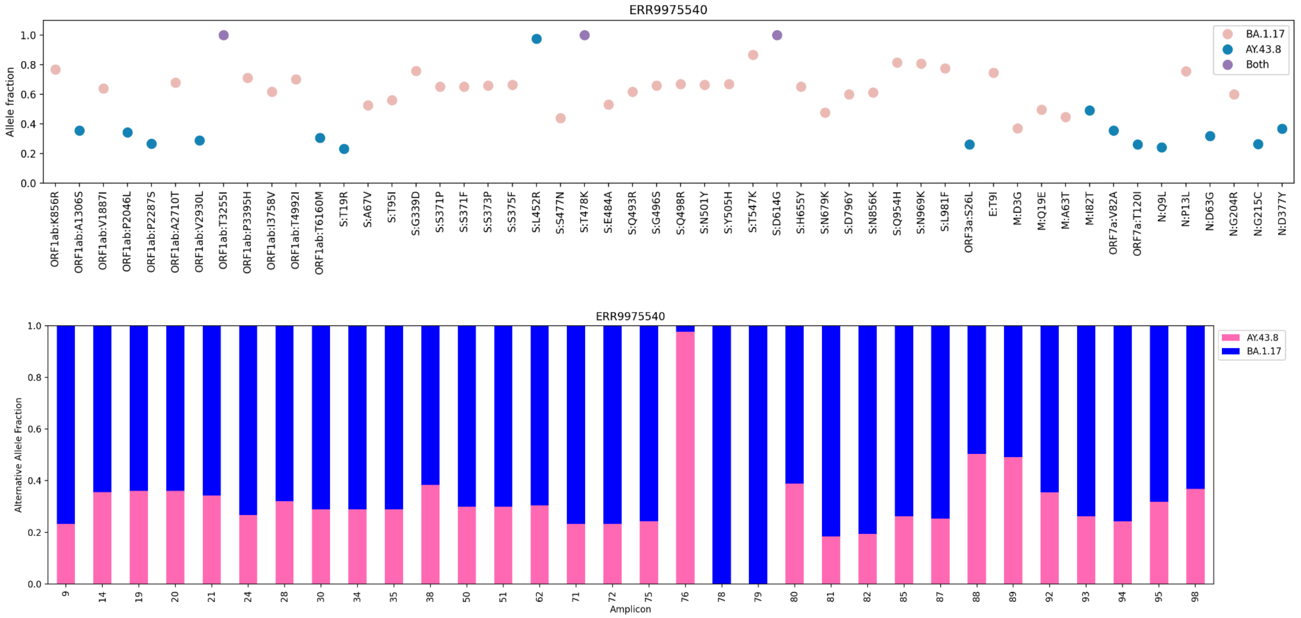
ERR9975540
Allele fraction
1.0
0.8
0.6
0.4
0.2
0.0
ORF1ab:K856R
ORF1ab:A1306S
ORF1ab:V1887I
ORF1ab:P2046L
ORF1ab:P2287S
ORF1ab:A2710T
ORF1ab:V2930L
ORF1ab:T3255I
ORF1ab:P3395H
ORF1ab:I3758V
ORF1ab:T4992I
ORF1ab:T6160M
S:T19R
S:A67V
S:T95I
S:G339D
S:S371P
S:S371F
S:S373P
S:S375F
S:L452R
S:S477N
S:T478K
S:E484A
S:Q493R
S:G496S
S:Q498R
S:N501Y
S:Y505H
S:T547K
S:D614G
S:H655Y
S:N679K
S:D796Y
S:N856K
S:Q954H
S:N969K
S:L981F
ORF3a:S26L
E:T9I
M:D3G
M:Q19E
M:A63T
M:I82T
ORF7a:V82A
ORF7a:T120I
N:Q9L
N:P13L
N:D63G
N:G204R
N:G215C
N:D377Y
BA.1.17
AY.43.8
Both
ERR9975540
Alternative Allele Fraction
1.0
0.8
0.6
0.4
0.2
0.0
9
14
19
20
21
24
28
30
34
35
38
50
51
62
71
72
75
76
78
79
80
81
82
85
87
88
89
92
93
94
95
98
Amplicon
AY.43.8
BA.1.17

E

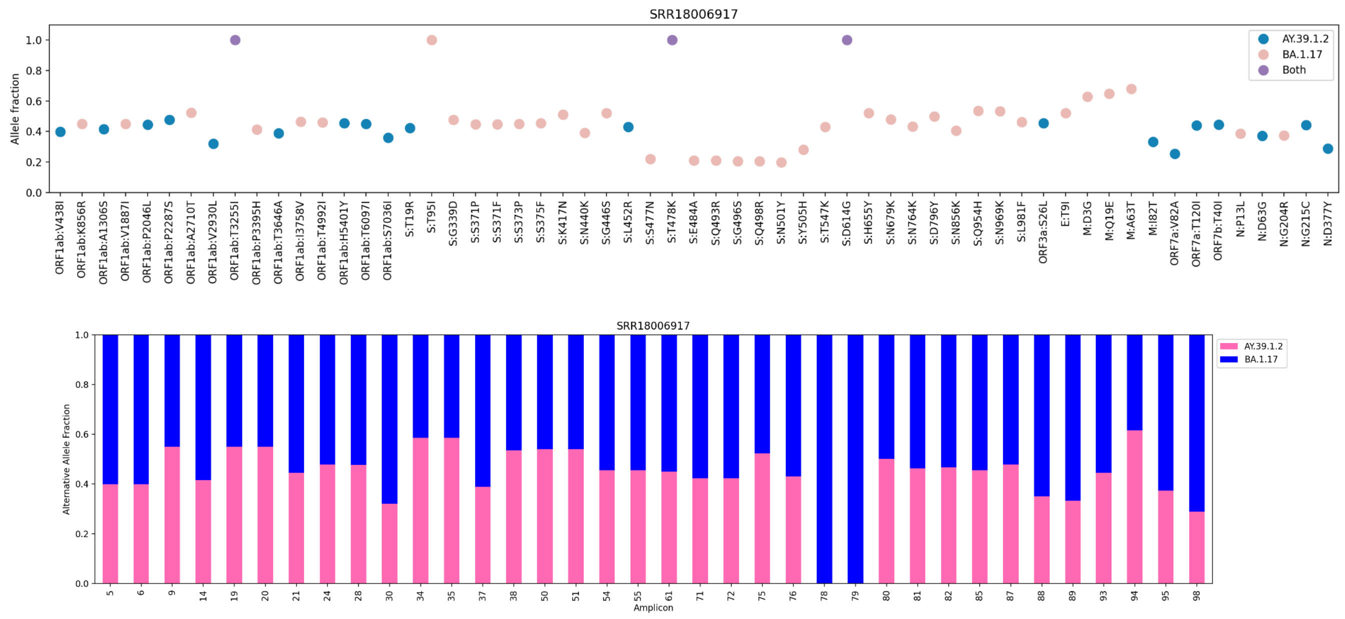

SRR18006917
Allele fraction
0.0
0.2
0.4
0.6
0.8
1.0
AY.39.1.2
BA.1.17
Both
ORF1ab:V438I
ORF1ab:K856R
ORF1ab:A1306S
ORF1ab:V1887I
ORF1ab:P2046L
ORF1ab:P2287S
ORF1ab:A2710T
ORF1ab:V2930L
ORF1ab:T3255I
ORF1ab:P3395H
ORF1ab:T3646A
ORF1ab:I3758V
ORF1ab:T4992I
ORF1ab:H5401Y
ORF1ab:T6097I
ORF1ab:S7036I
S:T19R
S:T95I
S:G339D
S:S371P
S:S371F
S:S373P
S:S375F
S:K417N
S:N440K
S:G446S
S:L452R
S:S477N
S:T478K
S:E484A
S:Q493R
S:G496S
S:Q498R
S:N501Y
S:Y505H
S:T547K
S:D614G
S:H655Y
S:N679K
S:N764K
S:D796Y
S:N856K
S:Q954H
S:N969K
S:L981F
ORF3a:S26L
E:T9I
M:D3G
M:Q19E
M:A63T
M:I82T
ORF7a:V82A
ORF7a:T120I
ORF7b:T40I
N:P13L
N:D63G
N:G204R
N:G215C
N:D377Y
SRR18006917
Alternative Allele Fraction
AY.39.1.2
BA.1.17
Amplicon
5 6 9 14 19 20 21 24 28 30 34 35 37 38 50 51 54 55 61 71 72 75 76 78 79 80 81 82 85 87 88 89 93 94 95 98

F

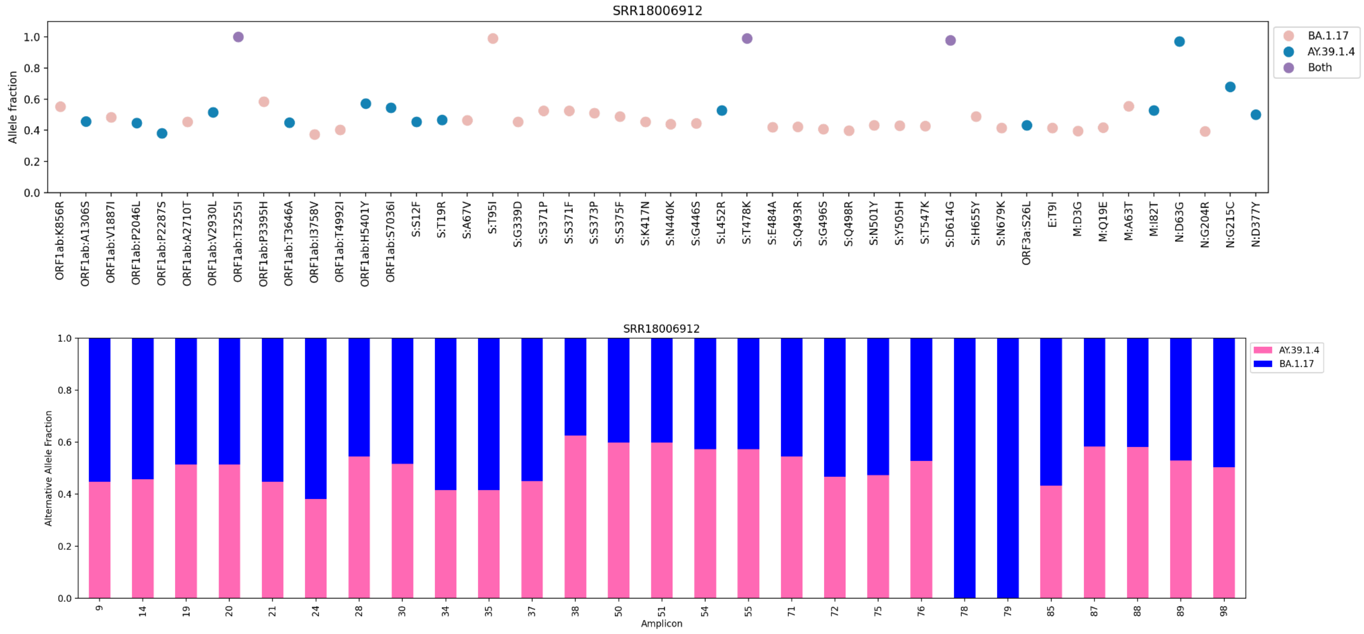

SRR18006912
Allele fraction
0.0
0.2
0.4
0.6
0.8
1.0
BA.1.17
AY.39.1.4
Both
ORF1ab:K856R
ORF1ab:A1306S
ORF1ab:V1887I
ORF1ab:P2046L
ORF1ab:P2287S
ORF1ab:A2710T
ORF1ab:V2930L
ORF1ab:T3255I
ORF1ab:P3395H
ORF1ab:T3646A
ORF1ab:I3758V
ORF1ab:T4992I
ORF1ab:H5401Y
ORF1ab:S7036I
S:S12F
S:T19R
S:A67V
S:T95I
S:G339D
S:S371P
S:S371F
S:S373P
S:S375F
S:K417N
S:N440K
S:G446S
S:L452R
S:T478K
S:E484A
S:Q493R
S:G496S
S:Q498R
S:N501Y
S:Y505H
S:T547K
S:D614G
S:H655Y
S:N679K
ORF3a:S26L
E:T9I
M:D3G
M:Q19E
M:A63T
M:I82T
N:D63G
N:G204R
N:G215C
N:D377Y
SRR18006912
Alternative Allele Fraction
AY.39.1.4
BA.1.17
Amplicon
9 14 19 20 21 24 28 30 34 35 37 38 50 51 54 55 71 72 75 76 78 79 85 87 88 89 98

Supplementary Table 1. Publicly available dataset with confirmed SARS-CoV-2 variants co-infection and their corresponding lineage assignment results using the Katmon pipeline.

Authors	SRA or EPI ISL	Sequencing platform	Publication report	Katmon report
Bolze et al. (2022)	SRR17951438	Illumina	Delta/Omicron co-infection	Delta AY.46.1/Omicron BA.1.1 co-infection
	SRR17951439	Illumina	Delta/Omicron co-infection	Delta AY.103/Omicron BA.1 co-infection
	SRR18272228	Illumina	Recombinant of Delta and Omicron BA.1	XS
	SRR18272229	Illumina	Recombinant of Delta AY.119 and Omicron BA.1.1	XS
	SRR18272230	Illumina	Co-infection of parental lineages Delta and Omicron, and Delta-Omicron recombinant lineage	Co-infection of Delta AY.103/Omicron BA.1.1/Recombinant XS
Dezordi et al. (2022)	EPI_ISL_1068258	Illumina	P.1/B.1.1.28 co-infection	P.1.1/B.1.1.274 co-infection
	EPI_ISL_2491769	Illumina	P.2/N.9 co-infection	P.2/N.9 co-infection
	EPI_ISL_2491781	Illumina	P.2/P.1 co-infection	P.2/P.1.1 co-infection
	EPI_ISL_2645599	Illumina	B.1.1.28/P.1 co-infection	B.1.1.28/P.1.1 co-infection
	EPI_ISL_2661789	Illumina	P.2/B.1.1.28 co-infection	P.2/B.1.1.28 co-infection
	EPI_ISL_2661931	Illumina	P.1/P.2 co-infection	P.1.12/P.2 co-infection
	EPI_ISL_2677092	Illumina	B.1.1.332/B.1.1.28 co-infection	B.1.1.332/P.1.10 co-infection
	EPI_ISL_2777552	Illumina	P.1/B.1.1.28 co-infection	P.1.12/B.1.1.28 co-infection
	EPI_ISL_3869215	Illumina	P.2/P.1 co-infection	P.2/P.1.1 co-infection
Garcia et al. (2024)	ERR12411105 and replicate ERR12411106	Illumina	Mixture of at least two recombinant strains, one AY.98.1-BA.5-AY.98.1 recombinant, and one BA.5-AY.98.1 recombinant	AY.98.1/B.1.1.529/BA.5.11 co-infection
Perez-Florido et al. (2023)	ERR9975540	Illumina	Delta/Omicron co-infection	Delta AY.43/Omicron BA.1.17 co-infection
	ERR9975546	Illumina	Omicron/Omicron (BA.1/BA.2) co-infection	Omicron BA.1/Omicron BA.2 co-infection
	ERR9975554	Illumina	Novel recombinant of Delta AY.98 annd Omicron BA.1.17	XS

	ERR9975555	Illumina	Novel recombinant of Omicron BA.2 and Omicron BA.1.1	XM
	ERR9975576	Illumina	Novel recombinant of Omicron BA.2 and Omicron BA.1.1	XM
Rockett et al. (2022)	SRR18006913	Illumina	Delta AY.39.1/Omicron B.1.1.529 co-infection	Delta/Omicron co-infection
	SRR18006914	Illumina	Delta AY.39.1/Omicron B.1.1.529 co-infection	Delta/Omicron co-infection
	SRR18006916	Illumina	Delta AY.39.1/Omicron BA.1 co-infection	Delta/Omicron co-infection
	SRR18006917	Illumina	Delta AY.39.1/Omicron BA.1 co-infection	Delta/Omicron co-infection
	SRR18006919	Illumina	Delta AY.39.1/Omicron BA.1 co-infection	Delta/Omicron co-infection
	SRR18006920	Illumina	Delta AY.39.1	AY.39.1.4
	SRR18006921	Illumina	Omicron BA.1	BA.1.17
	SRR18006923	Illumina	Delta AY.39.1/Omicron BA.1 co-infection	Delta/Omicron co-infection
	SRR18006924	Illumina	Delta AY.39.1/Omicron BA.1 co-infection	Delta/Omicron co-infection
	SRR18006912	ONT	Delta AY.39.1/Omicron BA.1 co-infection	Delta/Omicron co-infection
	SRR18006915	ONT	Delta AY.39.1/Omicron B.1.1.529 co-infection	Delta/Omicron co-infection
	SRR18006918	ONT	Delta AY.39.1/Omicron BA.1 co-infection	Delta/Omicron co-infection
	SRR18006925	ONT	Delta AY.39.1/Omicron BA.1 co-infection	Delta/Omicron co-infection

Supplementary Table 2. GISAID EPI Set of the 1078 retrospective samples analyzed with the pipeline.

Data Availability

GISAID Identifier: EPI_SET_251113ku

DOI: https://doi.org/10.55876/gis8.251113ku

All genome sequences and associated metadata in this dataset are published in GISAID's EpiCoV database. To view the contributors of each individual sequence with details such as accession number, Virus name, Collection date, Originating Lab and Submitting Lab and the list of Authors, visit EPI_SET_251113ku

Data Snapshot

EPI_SET_251113ku is composed of 1,078 individual genome sequences.

The collection dates range from 2021-07-31 to 2022-07-08;

Data were collected in 1 countries and territories.
